# Mapping tick-borne hazard across gradients of urban intensity in metropolitan regions

**DOI:** 10.1186/s13071-026-07448-4

**Published:** 2026-05-25

**Authors:** Wen Fu, Marie V. Lilly, Sung-Joo Lee, Heather Kopsco, Thilina Surasinghe, Maria Del Pilar Fernandez, Viorel Popescu, James Stark, Juanita Edwards, L. Hannah Gould, Patrick H. Kelly, Maria A. Diuk-Wasser

**Affiliations:** 1 Department of Ecology, Evolution, and Environmental Biology, Columbia University, New York, NY USA; 2 Department of Biological Sciences, Bridgewater State University, Bridgewater, MA USA; 3 Allen School for Global Health, Washington State University, Pullman, WA USA; 4 Global Vaccines Medical Affairs, Pfizer, Inc., Cambridge, MA USA; 5 Medical Enablement and Quality, Pfizer, Inc., Collegeville, PA USA; 6 Global Vaccines Medical Affairs, Pfizer, Inc., New York, NY USA; 7 United States Medical Affairs, Pfizer, Inc., Collegeville, PA USA

**Keywords:** Lyme disease hazard, *Ixodes scapularis*, Urban greenspaces, Landscape structure, Functional connectivity, Spatial modeling

## Abstract

**Background:**

Urban greenspaces are ecologically novel habitats where wildlife movement can shape vector-borne disease risk. Understanding how landscape structure influences tick presence and infection risk is essential for improving hazard assessment in metropolitan regions.

**Methods:**

We assessed Lyme disease hazard, defined as the density of *Borrelia*-infected *Ixodes scapularis* nymphs, across 141 greenspaces in New York City–Long Island (NYC–LI) and Greater Boston during 2023–2024. Sites were selected using a stratified design along gradients of housing density and wildlife functional connectivity. Active tick surveillance data were analyzed using spatial generalized linear mixed models that accounted for landscape composition, landscape configuration, and weather-related variables, as well as spatial autocorrelation. Continuous hazard maps were generated for each region.

**Results:**

Tick hazard declined with increasing housing density across both metropolitan regions. Percent tree canopy cover at the 100-m scale was positively associated with tick presence and nymph density, whereas percent impervious surface cover at the 1000-m scale showed a consistent negative association with hazard. Functional connectivity modified these relationships, with greenspaces embedded in more connected landscapes maintaining elevated hazard under higher levels of urbanization. Despite overall tick densities being lower in 2024, the spatial pattern of hazard in NYC–LI were similar between years, with higher hazard concentrated in eastern Long Island and central Staten Island. External validation in Greater Boston demonstrated comparable spatial patterns and good model performance, supporting cross-region transferability.

**Conclusions:**

Landscape composition and configuration jointly shape the distribution of tick vectors in urban environments. This standardized, reproducible, spatially explicit workflow advances tick hazard assessment in urban setting and, when integrated with human behavioral data, can improve exposure estimation and inform more targeted Lyme disease prevention.

**Graphical Abstract:**

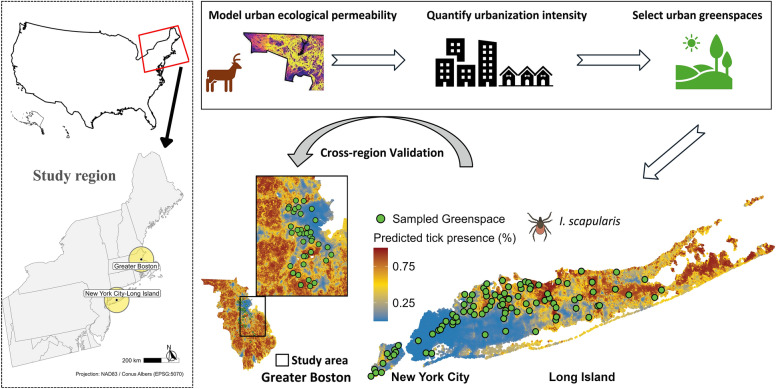

**Supplementary Information:**

The online version contains supplementary material available at 10.1186/s13071-026-07448-4.

## Background

As cities expand green infrastructure to enhance climate resilience, biodiversity, and human well-being in line with the United Nation’s Sustainable Development Goals, a critical yet often overlooked consequence is the shifting risk of zoonotic diseases in urban areas [1, 2]. Urban greenspaces ranging from parks and greenways to reforested corridors can create favorable conditions for wildlife hosts and vector species, supporting the persistence of enzootic transmission cycles [3, 4]. Among vector-borne diseases, those transmitted by mosquitoes and ticks have received the most attention, though research efforts vary considerably across regions, with urban tick-borne disease studies in the USA comparatively underrepresented relative to Europe, despite evidence of established risk in several US cities [4–6].

Lyme disease (LD), caused by *Borrelia burgdorferi* sensu lato spirochetal bacteria and transmitted by hard tick *Ixodes* spp. is the most reported tick-borne disease in the USA [7]. Despite ongoing efforts to manage tick vectors, reservoir hosts, and human exposure, LD remains a growing public health concern, with exposure potential continuing across an expanding range of *Ixodes* ticks [7–10]. Reported human cases have steadily increased over the past two decades, particularly in high-incidence regions such as the Northeast, mid-Atlantic, and upper Midwest, with recent surveillance data showing rising incidence rates ranging from 16.1 to 260 cases per 100,000 population in endemic areas [7].

The blacklegged tick (*Ixodes scapularis*), the primary vector of *B. burgdorferi* sensu lato in northeastern USA, has a 2 or 3-year life cycle comprising three blood-feeding stages (larva, nymph, and adult), each requiring a vertebrate host [11]. Nymphs are the principal stage responsible for human infections owing to their hard-to-detect small size and peak activity in late spring and early summer, which aligns with increased human outdoor activity [12]. Nymphs typically acquire *B. burgdorferi* during the larval stage by feeding on competent reservoir hosts such as white-footed mice (*Peromyscus leucopus*), eastern chipmunks (*Tamias striatus*), and northern short-tailed shrews (*Blarina brevicauda*) and some bird species [13, 14]. Environmental factors that support tick survival, including moderate temperatures and dense vegetation in deciduous forests that provide a humid substrate alongside the presence of reproductive hosts like white-tailed deer (*Odocoileus virginianus*), help sustain local *Ixodes* populations [15–17].

Urban landscapes consist of a patchwork of built, semi-natural, and natural spaces, creating ecological and social heterogeneity along the continuum from dense urban cores to less developed peripheries [18]. Multiscale patterns of landscape composition and configuration, from local to regional levels, influence host movement and human activity, contributing to spatial differences in tick-borne disease risks [19]. Although urban environments often support lower tick density than less-developed landscape, large human populations living around and using urban greenspaces can still create opportunities for encounter where infected *Ixodes* tick are present locally [20]. Despite growing awareness of tick-borne hazard in urban landscapes, existing studies face key limitations, including: (i) the lack of standardized metrics for urban gradients, which hinders meaningful comparisons across cities [21–24]; (ii) risk models that overlook multiscale urban landscape heterogeneity [25]; and (iii) a lack of predictive, spatially explicit maps that reflect tick-borne hazard across urban gradients [26].

Building on evidence that functional greenspace connectivity influences white-tailed deer movement and, together with local habitat characteristics, is associated with tick abundance [27, 28], this study translates these ecological relationships into spatially explicit maps of tick presence, density of nymph (DON), and density of *B. burgdorferi*-infected nymph (DIN). Using active tick surveillance data from 2023 to 2024 and multi-scale landscape metrics, we developed a transferable approach to characterize and map tick-borne hazard across urban gradients.

## Methods

### Study design and workflow

We applied this approach in two northeastern US metropolitan regions: five counties in New York City–Long Island (NYC–LI; Staten Island, Kings, Queens, Nassau, and Suffolk) and five counties in Greater Boston (Middlesex, Bristol, Suffolk, Norfolk, and Plymouth). To guide both sampling and analysis, we distinguished among three aspects of the landscape metrics: landscape composition, landscape configuration, and urban intensity. Landscape composition refers to the amount or proportion of a given land-cover type within or around a site such as vegetation cover or impervious surfaces [29]. Landscape configuration refers to how those habitat elements are spatially arranged across the surrounding landscape, including how connected or isolated habitat patches are [29, 30]. Urban intensity refers to the degree of human development surrounding a site and was represented here by housing unit density (HUD) [31].

**Fig. 1:**
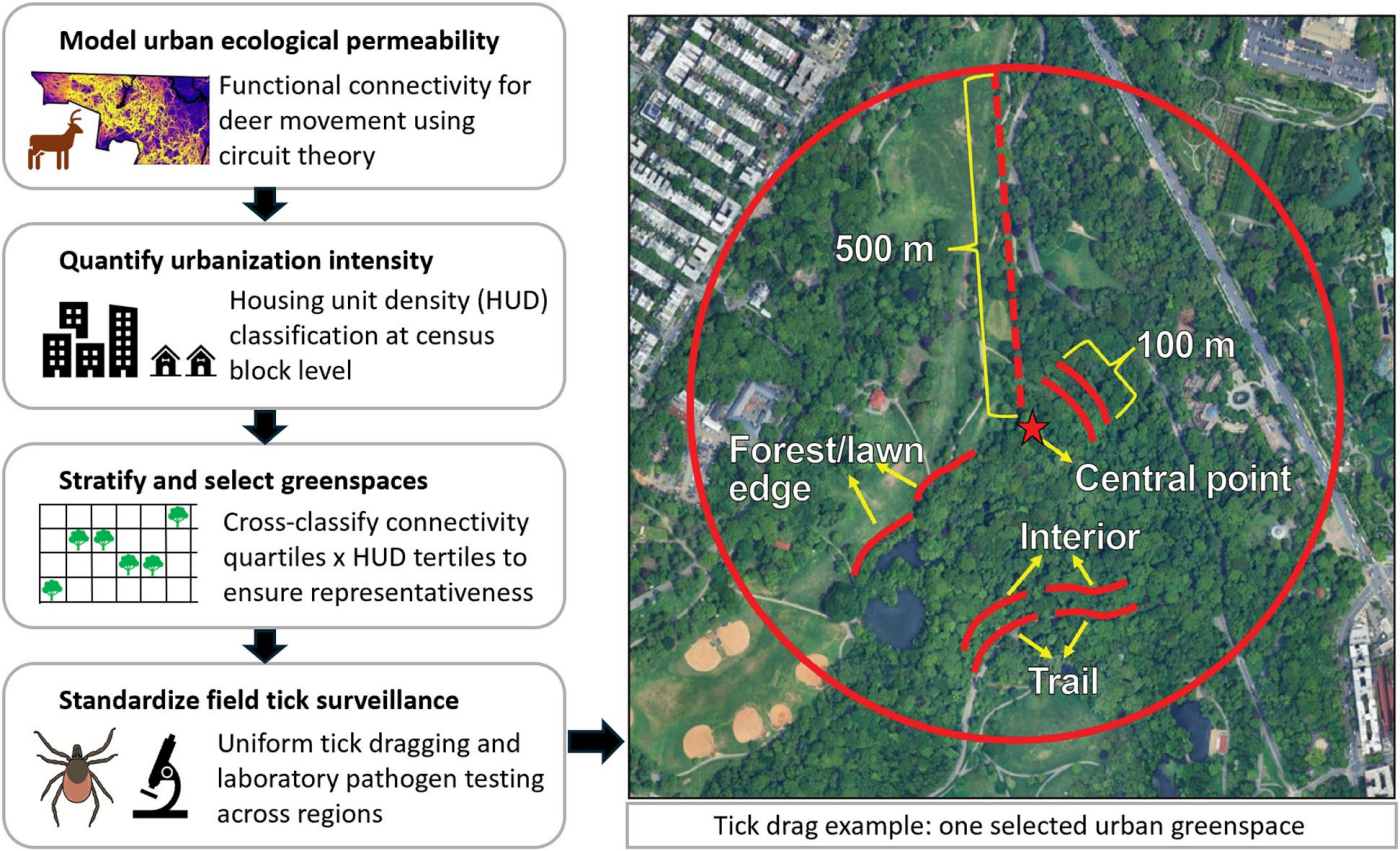
Workflow used for standardized tick hazard surveillance in urban areas

We used a stratified random design to select sampling greenspaces, stratified by landscape configuration (functional connectivity for white-tailed deer movement) and urban intensity (HUD); landscape composition variables, including percent impervious surface, were not used directly for greenspace stratification because impervious surface was highly correlated with HUD at the landscape scale [32]. By stratifying greenspace sample selection and standardizing field sampling, we ensure comparability across sites and years (Fig. 1).

### Functional connectivity for deer movement

To characterize landscape permeability (or the degree to which a landscape hinders or facilitates animal movement) for tick and pathogen distribution, we modeled functional connectivity for white-tailed deer using circuit theory implemented in Omniscape.jl [33]. Resistance surfaces were derived from land cover and infrastructure data, and mean current flow was calculated within 1000-m buffers surrounding each greenspace as a continuous connectivity metric (Additional file 1). This metric captures the connectedness of habitat across the surrounding landscape and with other greenspaces and was used as a proxy for potential host movement relevant to tick distribution. This metric was previously validated against seasonal deer occupancy data from Staten Island and Long Island [27].

### Urban intensity classification

Urban intensity was quantified using housing unit density (HUD; units/km^2^) calculated at the census block group (CBG) level from the 2023 American Community Survey [34]. Within each region, CBGs were stratified into tertiles representing low (≤ 375 units/km^2^), medium (376–1900 units/km^2^), and high (> 1900 units/km^2^) HUD strata, hereafter referred to as low-, medium-, and high-HUD strata. HUD was used to represent the degree of surrounding urban development at each greenspace.

### Stratified site selection by functional connectivity and urban intensity classes

Greenspaces were identified by merging publicly available conservation and recreational land databases (Additional file 1). Greenspaces were screened for eligibility on minimum size (≥ 5 ha), forest cover (≥ 20%), and sufficient trail length (≥ 800 m) to support standardized tick dragging [35]. Selected greenspaces were then assigned to a connectivity quartile on the basis of mean functional connectivity values and to an HUD strata on the basis of the surrounding CBG classification (Additional file 1). Final site selection followed a stratified random sampling design across combined connectivity quartiles and HUD tertiles.

### Standardized field sampling

In 2023, we surveyed 94 greenspaces across NYC–LI, spanning low- (*n* = 34), medium- (*n* = 39), and high-HUD strata (*n* = 21). In 2024, we resampled 45 NYC–LI greenspaces and added four new ones and surveyed 43 greenspaces in Greater Boston to support external model validation (Additional file 2: Supplementary Fig. S1–S2).

Tick sampling followed a standardized protocol known as “dragging” to collect nymphal blacklegged ticks (*I. scapularis*) [12]. Each greenspace was surveyed twice during the peak nymphal activity season. In NYC–LI, surveys were conducted from 23 May to 18 July 2023 and from 29 May to 18 June 2024 aligning with the typical seasonal peak. In Greater Boston, surveys began on 29 May 2024 with the first round conducted through 10 July. Owing to persistent rainfall, the second round extended into late August, beyond the typical activity window, and was excluded from the analysis to ensure seasonal consistency.

At each site, we aimed to sample up to 1600 m^2^ of forested habitat (approximately 800 m^2^ per survey [35]) within a 500-m radius of a central point, defined by either the greenspace centroid or 500 m from the greenspace entrance. Actual sampling area varied slightly across sites owing to local terrain, vegetation, and access constraints. Sampling targeted three habitat types including a minimum 200 m^2^ along trails, 200 m^2^ in interior forest (≥ 10 m from trails), 200 m^2^ at forest–lawn edges, and an additional 200 m^2^ in any of the three available habitat types. Ticks were collected from drag cloths every 10 m and preserved in either 85% ethanol or RNA/DNA Shield (Zymo Research, Irvine, CA). The global positioning system (GPS) coordinate points were recorded at the start of each transect and every 100 m thereafter.

Collected nymphs were morphologically identified under a dissecting microscope using standard taxonomic keys [36]. We screened up to 100 nymphal ticks for *B. burgdorferi* infection in each greenspace using multiplex reverse transcription quantitative polymerase chain reaction (RT–qPCR) [27].

### Statistical modeling framework

We developed three spatial generalized linear mixed models (GLMMs) to estimate: (i) the probability of nymph presence, (ii) density of questing nymphs (DON), and (iii) density of *Borrelia burgdorferi*-infected nymphs (DIN). Presence was modeled using a binomial GLMM with a logit link, while DON and DIN were modeled using negative binomial GLMMs. All models incorporated spatially structured random effects modeled as Gaussian processes with Matérn covariance functions to account for spatial autocorrelation and unmeasured environmental variation. Model equations and covariance specifications are provided in Additional file 3. Models were trained using 2023 NYC–LI data. Outcomes were defined at the transect level, and spatial predictions represent the expected outcome of a randomly placed transect within each grid cell.

### Predictor and buffer size selection

Environmental predictors were aggregated at three spatial scales (100 m, 500 m, and 1000 m) to capture local to broader-scale influences on tick vector, animal host, and pathogen distribution. Candidate predictors included metrics of landscape composition such as percent land cover, percent soil texture, percent impervious surface, and metrics of landscape configuration such as functional connectivity, and wildland–urban interface (WUI) (Table 1). Meteorological variables, including mean air temperature (AT) and vapor pressure deficit (VPD) during the sampling period months, were assigned on the basis of transect locations (Table 1).

**Table 1:** Candidate covariates evaluated in models predicting tick presence, density of nymphs (DON), and density of infected nymphs (DIN)

Covariate description	Hypotheses	Data source
Landscape composition metrics, constant in the 2 years of study 2023–2024		
Percent land cover including deciduous and coniferous forests, grassland, woody wetlands, and open water (%, continuous)	Suitable microclimatic conditions and habitat support tick survival and the presence of their hosts, potentially increasing tick abundance [15, 17]	National Land Cover Database (NLCD): land cover data derived from Landsat satellite imagery at a spatial resolution of 30 m [37]
Percent tree canopy cover (%, continuous)	Tree canopy maintains suitable microhabitats and positively associated with infected tick density [38]	USDA Forest Service: derived from multi-spectral satellite imagery at a spatial resolution of 30 m [39]
Percent impervious surface (%, continuous)	Greater impervious surface negatively correlates with tick abundance owing to reduced suitable habitat amount [27]	NLCD Imperviousness Dataset, 30-m resolution [40]
Percent soil texture: silt, clay, and sand (%, continuous)	Tick presence may be negatively associated with a high fraction of clay soil [41]	SoilGrids data: derived from machine learning models and soil observations, 250 m resolution [42]
Meteorological variables, varied in the 2 years of study 2023–2024		
Mean daily air temperature (°C, continuous)	Moderate temperatures during the active season are associated with increased nymphal activity, potentially elevating tick hazard [43–45]	PRISM Climate Data, 4 km [46]
Mean daily maximum vapor pressure deficit (VPD, %, continuous)	Higher VPD variation reflects drier conditions, often negatively associated with tick survival and activity [44, 45]	PRISM Climate Data, 4 km [46]
Landscape configuration metrics, constant in the 2 years of study 2023–2024		
Functional connectivity (categorical variable with thresholds < 50, 50–100, > 100)	Modeled greenspace connectivity is proxy for white-tailed deer movement and occupancy [27, 28]	Metrics derived from a published study with a spatial resolution at 30 m [27]
Wildland–urban interface: (categorical variable with three classes: non WUI, wildland–urban intermix and wildland–urban interface)	Zones where human development (e.g., housing infrastructure) intermixes with natural or semi-natural landscapes may create edge effects that are associated with increased tick hazard [47, 48]	USDA Forest Service Wildland–Urban Interface dataset, available at census-tract level [49]

All continuous predictors were standardized before analysis. Highly correlated variables were excluded, and the remaining predictors met variance inflation factor thresholds (variance inflation factor (VIF) < 4). For landscape composition predictors reflecting habitat amount, we identified the optimal spatial scale by fitting univariate spatial GLMMs across three buffer sizes (100 m, 500 m, and 1000 m) and selecting the scale with the lowest conditional Akaike information criterion (cAIC). As cAIC is based on the conditional likelihood, it evaluates model fit conditional on the estimated spatial random effects while accounting for model complexity [50]. Lower cAIC values indicate better model support. Predictors were subsequently included in multivariable models using their selected optimal scales.

### Model selection and validation

For each outcome, we began by fitting a global spatial model and containing all candidate predictors. We then applied a drop-one variable selection procedure to derive reduced models and selected the final model using cAIC comparisons (Additional file 3). We additionally evaluated interaction terms between percent impervious surface and functional connectivity to test whether the association between habitat amount and tick outcomes depended on landscape configuration.

Model performance was assessed through both internal and external validation. Internal validation used fivefold cross-validation. External validation involved applying models trained on 2023 NYC–LI data to independent 2024 datasets from NYC–LI and Greater Boston. For presence models, discrimination was evaluated using area under the receiver operating characteristic curve (AUC), while calibration and performance metrics for count models are reported in Additional file 3.

### Interannual comparison

To evaluate the consistency of tick occurrence patterns across the gradient of urban intensity between years, we compared DON and DIN within HUD strata (low, medium, high) among the 45 NYC–LI greenspaces sampled in both 2023 and 2024 using paired nonparametric tests.

### Spatial prediction and mapping

Final models were applied to a 100-m resolution grid covering the NYC–LI region to generate continuous prediction surfaces for tick presence, DON, and DIN. Environmental predictors were standardized using training-dataset parameters prior to prediction. All modeling and visualization were performed in R (version 4.4.2) using the spaMM, dplyr, tidyr, raster, ggplot2, and pROC packages [51].

## Results

### Nymphal tick sampling in 2023

**Fig. 2:**
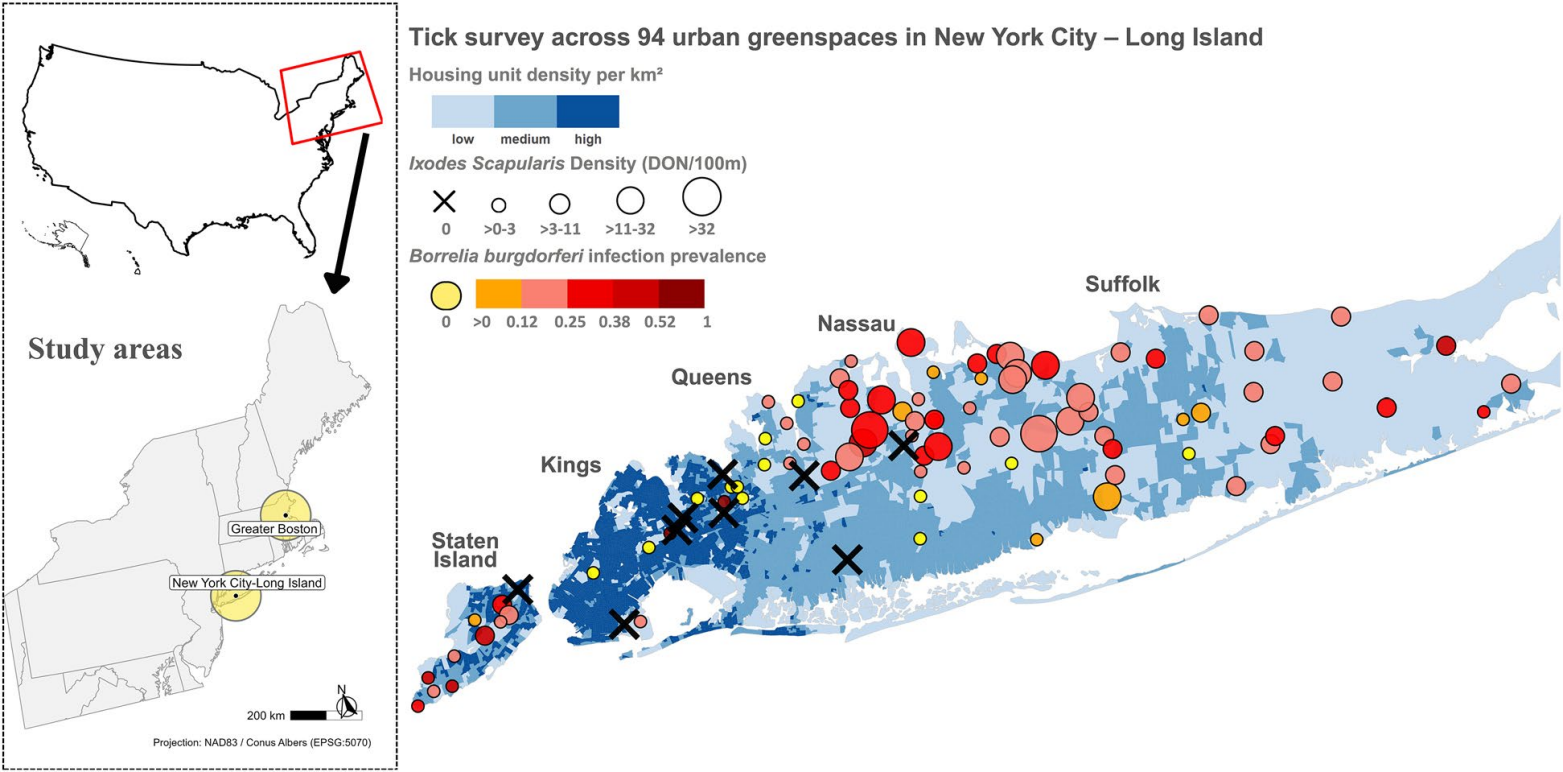
Map of 94 sampled greenspaces showing *Ixodes scapularis* nymphal density and *Borrelia burgdorferi *sensu lato (*Bb*sl) infection prevalence across the urban gradient in five counties of New York City–Long Island (NYC–LI; Staten Island, Kings, Queens, Nassau, and Suffolk), based on 2023 field surveys. Each greenspace is represented by a circle scaled according to nymphal density (DON, per 100 m^2^) and colored by the proportion of infected nymphs (*Bb*sl prevalence). Greenspaces where no nymphal tick were detected are indicated by black crosses. Urban intensity strata, represented by housing unit density (HUD) per km^2^ at the census-block level, were classified into low (< 375 units/km^2^), medium (375–1900 units/km^2^), and high (> 1900 units/km.^2^) categories and shaded from light- to dark-blue. Sampling locations and observed data for the five Greater Boston counties (Middlesex, Bristol, Suffolk, Norfolk, and Plymouth) are provided in Additional file 2: Supplementary Fig. S1 and Table S2

A total of 8221 nymphs were collected across 94 NYC–LI greenspaces in 2023, with 4148 individuals tested for *Borrelia burgdorferi* infection (Additional file 2: Supplementary Table S1). Figure 2 shows the spatial distribution of greenspace-level DON and infection prevalence across an urban gradient. Nymphs were detected at 82% of sampled greenspaces. Seventeen sites recorded fewer than three nymphs but greater than zero, including ten in high-HUD strata (47.6%) and seven in medium-HUD strata (17.9%). Of these, nine greenspaces yielded no nymphs, with six (28.6%) in high-HUD strata and three (7.7%) in medium-HUD strata.

Median DON and DIN declined with increasing HUD strata, with the highest values observed in low-HUD (DON = 6.71/100 m^2^; DIN = 1.54/100 m^2^), intermediate values in medium-HUD (DON = 2.97/100 m^2^; DIN = 0.56/100 m^2^), and the lowest values in high-HUD (DON = 0.19/100 m^2^; DIN = 0.00/100 m^2^) (Table 2). Despite these lower tick densities, infected nymphs were still detectable in several high-HUD greenspaces (Fig. 2; Additional file 2: Supplementary Tables S2 and S3).

**Table 2:** Tick distribution across the three urban intensity strata in New York City–Long Island, 2023

Urban intensity	Greenspaces	Average count	DON per 100 m^2^	DIN per 100 m^2^
		Median [IQR: Q1–Q3]		
Low HUD	34	88 [53–145]	6.71 [4.12–9.94]	1.54 [0.64–2.68]
Medium HUD	39	28 [9–95]	2.21 [0.61–6.30]	0.37 [0.06–1.79]
High HUD	21	3 [0–11]	0.19 [0–0.85]	0 [0–0.14]

*IQR* interquartile range

Urban intensity strata, represented by housing unit density (HUD) per km^2^ at the census-block level, was classified into low (< 375 units/km^2^), medium (375–1900 units/km^2^), and high (> 1900 units/km^2^) categories. The average count refers to the number of nymphal *Ixodes scapularis* collected in each urban greenspace. DON represents the number of nymphal *I. scapularis* per 100 m^2^, while DIN indicates the number of *Borrelia*-infected nymphs per 100 m^2^

### Predictor and buffer size selection

Across univariate models, landscape composition and configuration predictors showed generally consistent directional associations with tick presence across buffer sizes (Additional file 4: Supplementary Table S4). Percent tree canopy cover, percent deciduous forest, percent sand content, and functional connectivity metric were positively associated with tick presence, whereas percent impervious surface consistently showed negative associations. Percent evergreen forest, percent woody wetlands, and percent open water were not significant predictors at any spatial scale. Percent grassland was negatively associated with tick presence at the 100-m scale, with weaker and nonsignificant associations at broader scales.

Conditional AIC comparisons identified optimal buffers of 100 m for percent tree canopy cover and percent deciduous forest, 500 m for percent woody wetlands, and 1000 m for percent impervious surface, functional connectivity metric, and percent soil sand (Additional file 4: Supplementary Table S4). Among fixed-scale predictors, mean AT was negatively associated with tick occurrence, while VPD and WUI classification showed no significant associations (Additional file 4: Supplementary Table S5).

### Model selection and internal validation (2023)

Across all three spatial GLMMs, percent tree canopy cover and percent impervious surface emerged as the strongest predictors, with their removal producing the largest declines in model fit during the drop-one selection procedure (Additional file 4: Supplementary Tables S8 and S12). The optimal models for tick presence and DIN did not retain interaction terms, whereas the optimal DON model included an interaction between percent impervious surface and categorical functional connectivity (Table 3). Results for all global model specifications are provided in Additional file 4: Supplementary Tables S6–S13.

**Table 3:** Optimal model used to predict the presence, overall density (DON), and infected density (DIN) of questing *Ixodes scapularis* nymphs, New York City–Long Island, NY, USA, 2023

Predictor	Buffer (m)	Presence coeff	DON Coeff	DIN Coeff
Continuous variables				
% Tree canopy cover	100	0.60^**^	0.38^*^	0.10
% Impervious surface	1000	–0.89^**^	–1.25^*^	–0.54^*^
Categorical: connectivity				
Medium versus Low	1000	–	0.25	0.19
High versus low	1000	–	0.53	0.5
Interactions				
Medium connectivity × impervious	1000	–	0.59^*^	–
High connectivity × impervious	1000	–	0.59	–

Asterisks symbols denote statistically significant predictors with (^**^) for an*P*-value < 0.001 and (^*^) for an*P*-value < 0.05. Results are from the final optimal models with (i) a binomial model for nymph presence, (ii) a negative binomial model for total nymph density (DON), and (iii) a negative binomial model for *Borrelia burgdorferi*-infected nymph density (DIN). Continuous variables are standardized and assessed at spatial buffers optimized by model fit (percent tree canopy cover: 100 m; percent impervious surface: 1000 m). Functional connectivity is a categorical variable based on threshold values (low: < 50, medium: 50–100, high: > 100) derived from landscape resistance surfaces validated against white-tailed deer occupancy data [27]. Interaction terms were used to assess  whether the association between percent impervious surfaces and tick density varied by connectivity class

Grouped fivefold cross-validation indicated stable predictive performance across alternative presence thresholds (AUC ≈ 0.81; Additional file 5: Supplementary Fig. S4). Model diagnostics showed no evidence of overdispersion, residual spatial autocorrelation, or heteroskedasticity (Additional file 5: Supplementary Fig. S7).

### Predictors of tick presence, DON, and DIN

In multivariable models, percent impervious surface was a consistent negative predictor of tick presence, DON, and DIN (Table 3). Tick presence was strongly positively associated with percent tree canopy cover within 100 m (coefficient = 0.60, an*P* < 0.001) and negatively associated with percent impervious surface within 1000 m (coefficient = −0.89, an*P* < 0.001).

For DON, percent tree canopy cover remained positively associated (coefficient = 0.38, an*P* < 0.05), and percent impervious surface showed a strong negative effect (coefficient = −1.25, an*P* < 0.05). The negative impact of percent impervious surface on predicted DON was amplified at low connectivity sites, such that predicted DON was the lowest at high impervious, low connectivity greenspaces (Additional file 4: Supplementary Fig. S3).

In the DIN model, percent impervious surface remained a significant negative predictor (coefficient = −0.54, an*P* < 0.05), while percent tree canopy cover and interaction terms were not statistically significant.

### Predicted spatial distribution of tick hazard in NYC–LI (2023)

**Fig. 3:**
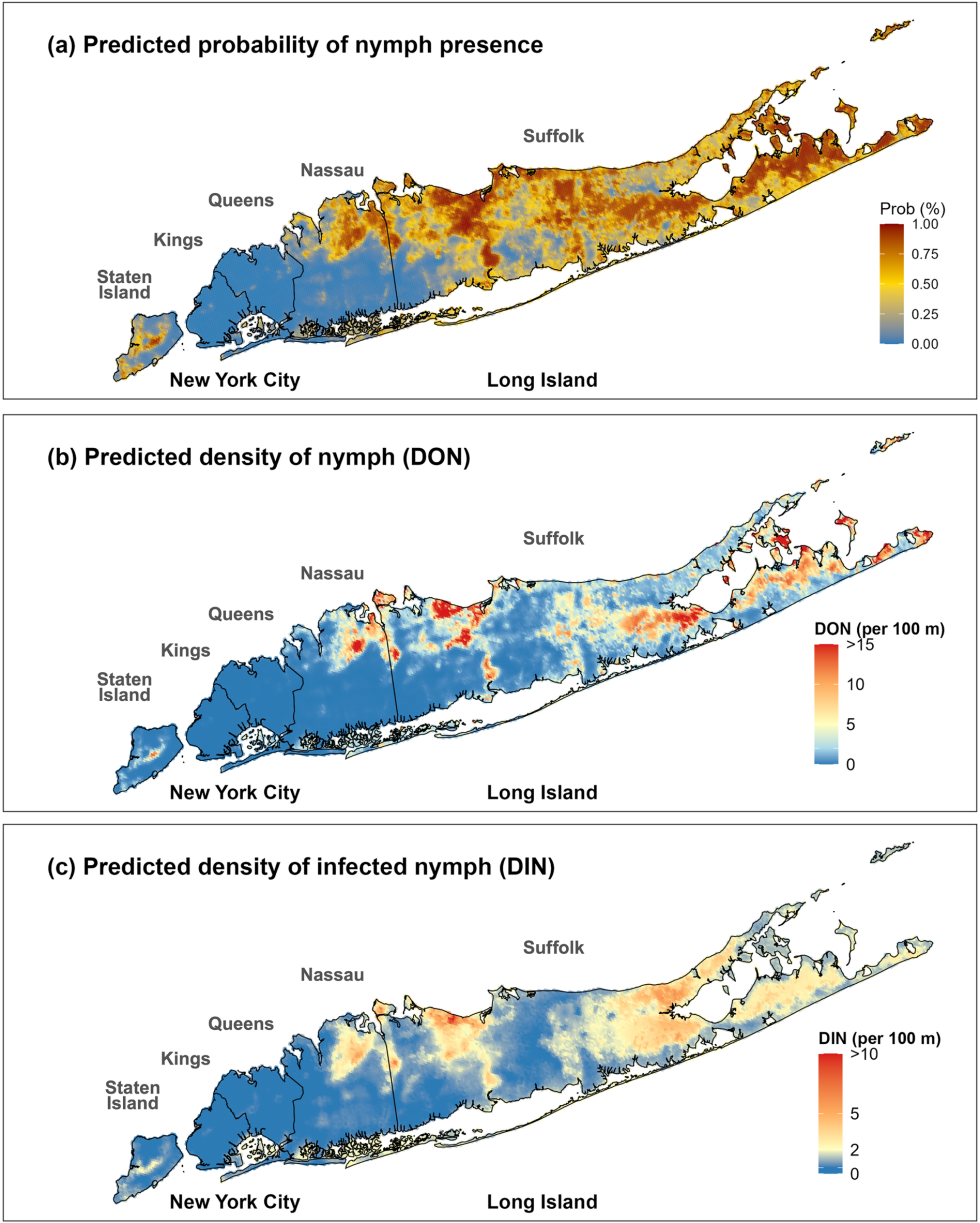
Predicted tick distribution across five counties in New York City–Long Island, 2023. **a** Predicted probability of nymphal *Ixodes scapularis* presence; **b** Predicted density of *I. scapularis* nymphs (DON, per 100 m); **c** Predicted density of *Borrelia burgdorferi*-infected nymphs (DIN, per 100 m). Predictions were generated using spatial GLMMs applied to a 100-m grid across the study area

Predicted maps showed consistent spatial patterns across all outcomes (Fig. 3a–c). The probability of nymph presence was the highest in central Staten Island and across much of Long Island, particularly in parts of Nassau county and most of Suffolk county, and the lowest in highly urbanized areas of NYC, including Kings and Queens counties (Fig. 3a). The predicted distribution was right-skewed, with a median probability of approximately 0.19 (Additional file 5: Fig. S5).

Predicted DON and DIN closely mirrored presence patterns, with elevated values concentrated in northern Nassau, central-to-eastern Suffolk, and central Staten Island (Fig. 3b and c). Predicted DON values exceeding 15 nymphs per 100 m were primarily confined to eastern Long Island, while predicted DIN values above 5 per 100 m were most prominent in central Suffolk and parts of northern Nassau. In contrast, predicted tick densities remained similarly low across Kings and Queens counties and moderate in Staten Island.

### Nymphal tick sampling in 2024 and interannual comparison

In Greater Boston, round 1 sampling (29 May to 10 July) yielded 570 nymphs, while round 2 (9 July to 27 August) yielded only 68 nymphs from 43 greenspaces (Additional file 2: Supplementary Table S2). Persistent rainfall shifted the second survey round to dates beyond nymphal activity period, so they were excluded from the analysis. In addition, one site was excluded owing to only being sampled at round 2. Thus, tick data from 42 sites sampled during round 1, with an overall *Borrelia* infection prevalence of 19.6%, were used for model validation. In 2024, 1080 nymphs were collected from 49 NYC–LI greenspaces, all of which were tested for *Borrelia* infection, yielding an overall infection prevalence of 23.4% (Additional file 2: Supplementary Table S3).

Tick counts, DON, and DIN consistently declined with increasing housing unit density in both NYC–LI and Boston in 2024 (Table 4). In NYC–LI, low-HUD greenspaces had the highest median tick counts and hazard metrics, while high-HUD sites showed a small number less than three nymphs. A similar pattern was observed in Boston’s first survey round, with notably reduced tick presence and densities in highly urbanized areas.

**Table 4:** Tick distribution along three urban intensity strata in New York City–Long Island and Greater Boston, 2024

Urban gradient	Number of greenspaces	Average count	DON per 100 m^2^	DIN per 100 m^2^
		Median (IQR: Q1–Q3)		
New York				
Low HUD	18	37 [17–54]	2.31 [1.06–3.18]	0.42 [0.16–1.27]
Medium HUD	22	14 [5.25–36.75]	0.86 [0.33–2.45]	0.35 [0.13–0.75]
High HUD	9	1 [0–2.75]	0.06 [0–0.15]	0.03 [0–0.13]
Boston (round 1^*^)				
Low HUD	13	17 [6–40]	1 [0.13–1.60]	0.17 [0.01–0.38]
Medium HUD	19	5 [2–14]	0.17 [0–0.43]	0.02 [0–0.10]
High HUD	10	0 [0–3.5]	0 [0–0]	0 [0–0]

Urban intensity strata, represented by housing unit density (HUD) per km^2^ at the census-block level, was classified into low (< 375 units/km^2^), medium (375–1900 units/km^2^), and high (> 1900 units/km^2^) categories. The average count refers to the number of nymphal *Ixodes scapularis* collected in each urban greenspace per season. DON represents the number of nymphal *I. scapularis* per 100 m^2^, while DIN indicates the number of *Borrelia*-infected nymphs per 100 m^2^

^*^In Greater Boston, surveys began on 29 May 2024, with the first round conducted through 10 July. Owing to persistent rainfall, the second round extended into late August, beyond the typical activity window, and was excluded from the analysis to ensure seasonal consistency

**Fig. 4:**
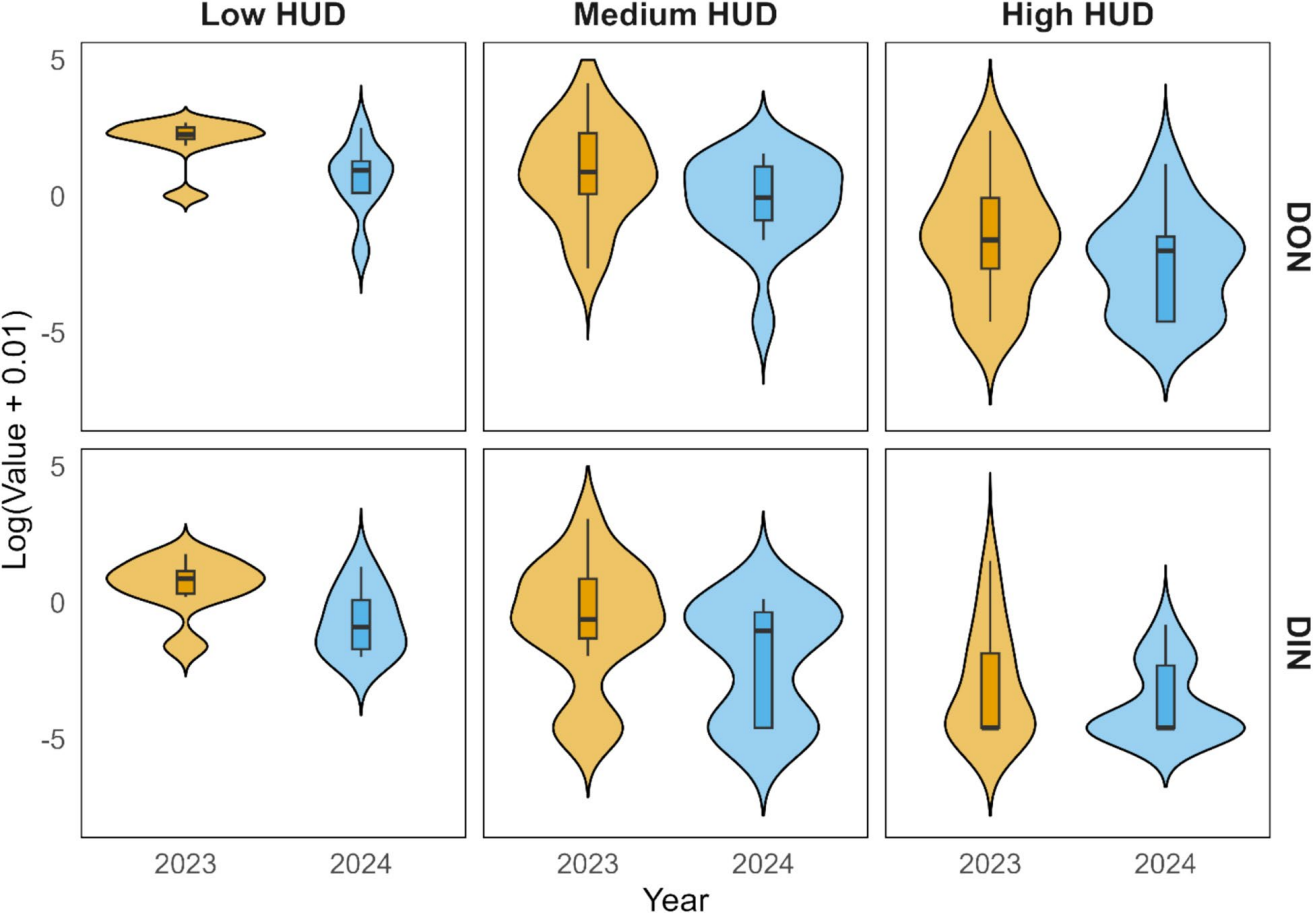
Distribution of log-transformed nymph density (DON, top) and infected nymph density (DIN, bottom) across the urban intensity strata (low, medium, high) among 45 greenspaces in New York City–Long Island in both 2023 and 2024. Boxes represent interquartile ranges, with horizontal lines indicating medians

Paired analyses of 45 NYC–LI sites sampled in both 2023 and 2024 showed that DON was significantly lower in 2024 across all HUD categories (low, medium, and high; all an*P* < 0.05). DIN also declined significantly among greenspaces in low- and medium-HUD strata but did not differ significantly in high-HUD strata. Despite year-to-year variation in absolute densities, the relative urban gradient in hazard (low > medium > high HUD) remained consistent across years (Fig. 4).

### External model validation and transferability (2024)

When applied to independent 2024 datasets, the presence model trained using NYC–LI data showed moderate discrimination performance for both NYC–LI (AUC = 0.79; Additional file 5: Supplementary Fig. S8) and Greater Boston (AUC = 0.77; Additional file 5: Supplementary Fig. S9). Calibration analyses indicated good discrimination but some overconfidence in predicted probabilities (slope < 1). Spatial prediction maps for 2024 showed broadly similar patterns across NYC–LI and Greater Boston (Additional file 5: Supplementary Fig. S10), with higher predicted presence in low- and medium-HUD strata and lower predicted presence in highly urbanized core areas.

## Discussion

This study presents a standardized, spatially explicit workflow to assess Lyme disease hazard in urban ecosystems, applied in New York City–Long Island (NYC–LI) and Greater Boston. By integrating stratified site selection, standardized field surveillance, and spatial generalized linear mixed models, we mapped tick hazard while accounting for landscape composition, landscape configuration, and weather-related predictors across both regions.

Across all three outcome models, percent impervious surface within a 1000-m buffer consistently emerged as a key landscape predictor of tick presence, DON, and DIN, underscoring the importance of habitat amount in shaping tick vector distributions [52]. Though forest fragments in highly impervious urban areas can retain conditions that harbor infected ticks, including the presence of competent pathogen reservoir such as the white-footed mouse [53], the observed decrease in hazard with increasing percent impervious surface (and decreasing percent forest cover) supports the existence of a limiting habitat amount required for tick and pathogen persistence. This pattern highlights an important distinction between habitat amount and habitat fragmentation [54–57]. While other studies have proposed that increased human-driven fragmentation, in rural or urban settings, can elevate tick-borne disease hazard and human exposure to infected tick [54, 55, 58], these studies do not account for threshold levels of habitat amount needed for tick and pathogen persistence. By comparison, the highly urban nature of this NYC study demonstrates how increased urbanization can eventually reduce habitat amount below the threshold level required and lead to the failure of ticks and pathogens establishment despite potentially high abundance of highly competent hosts [53]. Similarly, tick extinctions were found to be frequent in small forest fragments across multiple northeastern US states; the land cover between fragments was not reported [56].

A more nuanced understanding arises when accounting for landscape configuration, beyond habitat amount alone. While previous studies have focused on simple metrics of fragmentation such as patch size and isolation [54, 55], our study shows that functional habitat connectivity for white-tailed deer and other wildlife movement is a more biologically meaningful metric, in particular in urban areas with limited habitat amount [27, 28, 52, 59]. At high impervious cover, greater connectivity increased DON by facilitating host movement and reducing patch isolation. Conversely, at lower impervious cover, increased connectivity was associated with lower hazard, consistent with habitat fragmentation elevating the hazard by favoring more competent host communities, i.e., a “dilution effect” [52, 56, 60]. Overall, these findings suggest that functional connectivity for wildlife (here represented by white-tailed deer movement) can modulate the relationship between habitat amount and tick hazard in urban landscapes, emphasizing the need to consider both landscape composition and configuration when assessing tick habitat suitability [18, 27, 52].

Tree canopy cover within 100-m buffers surrounding transect-level sampling location within greenspaces was positively associated with both tick presence and DON. Prior studies have shown that *Ixodes scapularis* survival is higher in forested and edge habitats than in open habitats, because local temperature and vapor pressure deficit are too high or too dry, and that canopy cover and leaf litter buffer near-ground microclimate experienced by ticks [16, 61, 62]. Consistent with this, percent tree canopy cover in our study likely captured these fine-scale microclimatic conditions more directly than weather variables. Although air temperature showed significant negative effect with nymph occurrence in univariate analyses, it was not retained in the final model. This suggests that weather variables may capture broad seasonal or regional variation in tick-favorable conditions but their apparent effects are reduced once local habitat amount is included. More generally, reviews of *I. scapularis* tick ecology note that while temperature and humidity are biologically important for tick survival and host-seeking behavior, their statistical associations with local abundance are often inconsistent across studies and spatial scales, likely because local habitat conditions become increasingly important at finer spatial resolution [62, 63]. Nevertheless, neither percent tree canopy cover nor functional connectivity significantly predicted DIN, suggesting that the density of infected nymphs may depend less on habitat amount alone and more on ecological processes not directly measured here, including pathogen host dynamics [18, 64, 65].

Urban environments are novel ecosystems in which habitat amount, habitat fragmentation, host movement, and heterogeneous human settlement interact to shape tick vector distribution and pathogen transmission [66]. Though DON and DIN declined in 2024 compared with 2023 (NYC–LI), the underlying trends (i.e., rankings) from low to high urban intensity strata remain consistent. This temporal consistency suggests that urban tick hazard is structured by broad landscape composition and configuration measured in this study, even when absolute tick abundance varies between years.

Spatial predictions from the final models revealed pronounced heterogeneity in tick hazard, with hotspots of high DON and DIN on Staten Island, Nassau, and Suffolk counties consistent with prior surveillance reports [28, 67, 68]. These findings underscore that urban tick hazard is spatially clustered rather than uniformly distributed across greenspaces. The resulting high-resolution (100-m) hazard maps provide a practical tool for urban public health planning by identifying areas where targeted intervention such as vegetation management, host deterrence, or enhanced surveillance may be prioritized. [60]. Moreover, this study provides a standardized workflow that can be replicated in other metropolitan regions and reapplied over time to support comparable tick hazard assessment.

Several limitations should be considered. First, DON and DIN are count-based outcomes that can vary substantially over short distances, especially in patchy urban greenspaces. Accordingly, model outputs should be interpreted primarily as indicators of relative spatial variation rather than precise absolute densities. Second, DIN was more difficult to model than the other hazard metrics because it depends not only on tick density but also pathogen circulation in reservoir hosts, which was not measured directly in this study. Third, rainfall-limited sampling in Boston may have influenced data quality and model fit. Moreover, our sampling frame, although stratified, covered a limited share of all urban census block groups and may underrepresent extremely fragmented or highly connected areas. Lastly, while our connectivity metric accounted for broad landscape permeability, smaller anthropogenic barriers (e.g., fences, walls, roads) were not included. Future studies could improve DON and DIN prediction by incorporating finer-scale habitat and management variables, direct data on host abundance or reservoir communities, more detailed resistance surfaces, and repeated sampling over additional years.

## Conclusions

This study provides a replicable and ecologically grounded standardized workflow for quantifying and mapping urban tick hazards. Our results demonstrate that *Ixodes scapularis* nymphs and *Borrelia*-infected nymphs can occur even in highly urbanized core areas, particularly where habitat connectivity supports host access. Integrating such spatial hazard models with behavioral and demographic data will support more accurate exposure assessments and guide targeted interventions in urban and peri-urban settings.

## Supplementary Information

Supplementary Material 1.

Supplementary Material 2.

Supplementary Material 3.

Supplementary Material 4.

Supplementary Material 5.

## Data Availability

Aggregated tick sampling data and pathogen infection prevalence at the greenspace level are provided in Additional File 2.Data supporting the main conclusions of this study are included in the manuscript.
