# Supplementary material for: Mapping tick-borne hazard across gradients of urban intensity in metropolitan regions
Source: Parasit Vectors. 2026 May 25;19:295. doi: 10.1186/s13071-026-07448-4 (PMC13386969; doi:10.1186/s13071-026-07448-4)
Supplement: Supplementary file 1 — Supplementary Material 1. [file 13071_2026_7448_MOESM1_ESM.docx]

**Mapping tick-borne hazard across gradients of urban intensity in metropolitan regions**

Wen Fu^1*^, Marie V. Lilly^1^, Sung-Joo Lee^1^, Heather Kopsco^1^, Thilina Surasinghe^2^, Maria Del Pilar Fernandez^3^, Viorel Popescu^1^, James Stark^4^, Juanita Edwards^5^, L. Hannah Gould^6^, Patrick H. Kelly^7^, Maria A. Diuk-Wasser^1^

1. Department of Ecology, Evolution, and Environmental Biology, Columbia University, New York, NY, USA

2. Department of Biological Sciences, Bridgewater State University, Bridgewater, MA, USA

3. Allen School for Global Health, Washington State University, Pullman, WA, USA

4. Global Vaccines Medical Affairs, Pfizer, Inc., Cambridge, MA, USA

5. Medical Enablement and Quality, Pfizer, Inc., Collegeville, PA, USA

6. Global Vaccines Medical Affairs, Pfizer, Inc., New York, NY, USA

7. United States Medical Affairs, Pfizer, Inc. Collegeville, PA, USA

Corresponding author: Wen Fu, [wf2317@columbia.edu](mailto:wf2317@columbia.edu)

## **Additional file 1: Stratified sampling site selection in New York City-Long Island (NYC-LI)**

**Landscape connectivity modeling**

To guide ecological site selection across a gradient of landscape permeability, we modeled functional connectivity for white-tailed deer movement across New York City and Long Island using Omniscape.jl, a circuit theory–based modeling framework that simulates animal movement across resistance surfaces informed by land cover and infrastructure features [1,2]. Model outputs were validated using camera trap data collected from 44 sites on Staten Island and Long Island during 2022–2023 [3]. Validation analyses showed that connectivity scores, measured as mean current flow, were significantly associated with deer detections within a 1,000-meter buffer surrounding each site [3].

Based on this validation and the ecological relevance of the 1,000-m scale to urban white-tailed deer home range size [4], we calculated mean current flow within a 1,000-m buffer surrounding all publicly accessible parks in New York City-Long Island. Parks were then classified into four connectivity categories based on the distribution of current flow values: low (0.3-21.9), medium-low (21.9-67.6), medium-high (67.6-143.5), and high (143.5-532.4). Stratified random sampling within each of these four connectivity categories was used to ensure that selected sites represented the full range of landscape permeability and to minimize geographic clustering.

**Greenspace eligibility and stratified site selection**

Candidate greenspaces were identified by merging spatial layers from five publicly available databases: (1) U.S. Protected Areas Database [5], (2) New York State Conservation Lands, (3) New York Natural Heritage Program, (4) MassGIS Protected and Recreational Space [6], and (5) the National Conservation Easement Database [7]. Greenspaces were retained if they met the following criteria: ≥5 ha in size, ≥20% forest cover, publicly accessible, and containing ≥800 m of trail to support standardized tick dragging [8].

From this pool of eligible greenspaces, we selected sites using a stratified random sampling design based on two key metrics: greenspace functional connectivity and housing unit density (HUD). Each greenspace was first assigned to one of four connectivity quantiles based on its mean current flow value. We then aggregated surrounding census blocks within concentric 1- to 5-km buffers and assigned each site to the dominant HUD strata (low, medium, or high). Finally, sites were randomly selected across the combined connectivity–HUD strata to ensure broad representation of ecological and socio-environmental conditions along the urban gradient.

**References**

1. Landau V, Shah V, Anantharaman R, Hall K. Omniscape.jl: Software to compute omnidirectional landscape connectivity. J Open Source Softw. The Open Journal; 2021;6:2829. https://doi.org/10.21105/joss.02829

2. Mcrae B, Popper K, Jones A, Schindel M. Conserving Nature’s Stage: Mapping Omnidirectional Connectivity for Resilient Terrestrial Landscapes in the Pacific Northwest. 2016; https://doi.org/10.13140/RG.2.1.4158.6166

3. Lilly M V., Davis M, Kross SM, Konowal CR, Gullery R, Lee SJ, et al. Functional connectivity for white-tailed deer drives the distribution of tick-borne pathogens in a highly urbanized setting. Landsc Ecol [Internet]. Springer Science and Business Media B.V.; 2025 [cited 2025 May 26];40. https://doi.org/10.1007/S10980-025-02101-4,

4. VanAcker MC, Little EAH, Molaei G, Bajwa WI, Diuk-Wasser MA. Enhancement of risk for lyme disease by landscape connectivity, New York, New York, USA. Emerg Infect Dis. Centers for Disease Control and Prevention (CDC); 2019;25:1136–43. https://doi.org/10.3201/eid2506.181741

5. PAD-US Data [Internet]. 2022 [cited 2025 Jun 17]. https://www.usgs.gov/programs/gap-analysis-project/science/pad-us-data-download. Accessed 17 Jun 2025

6. MassGIS Data [Internet]. 2025 [cited 2025 Jun 17]. https://www.mass.gov/info-details/massgis-data-protected-and-recreational-openspace. Accessed 17 Jun 2025

7. NCED [Internet]. 2025 [cited 2025 Jun 17]. https://www.conservationeasement.us/. Accessed 17 Jun 2025

8. Diuk-Wasser MA, Gatewood AG, Cortinas MR, Yaremych-Hamer S, Tsao J, Kitron U, et al. Spatiotemporal Patterns of Host-Seeking Ixodes scapularis Nymphs (Acari: Ixodidae) in the United States. J Med Entomol [Internet]. Oxford Academic; 2006 [cited 2025 Jun 21];43:166–76. https://doi.org/10.1093/JMEDENT/43.2.166
