# Supplementary material for: Mapping tick-borne hazard across gradients of urban intensity in metropolitan regions
Source: Parasit Vectors. 2026 May 25;19:295. doi: 10.1186/s13071-026-07448-4 (PMC13386969; doi:10.1186/s13071-026-07448-4)
Supplement: Supplementary file 2 — Supplementary Material 2. [file 13071_2026_7448_MOESM2_ESM.docx]

**Mapping tick-borne hazard across gradients of urban intensity in metropolitan regions**

Wen Fu^1*^, Marie V. Lilly^1^, Sung-Joo Lee^1^, Heather Kopsco^1^, Thilina Surasinghe^2^, Maria Del Pilar Fernandez^3^, Viorel Popescu^1^, James Stark^4^, Juanita Edwards^5^, L. Hannah Gould^6^, Patrick H. Kelly^7^, Maria A. Diuk-Wasser^1^

1. Department of Ecology, Evolution, and Environmental Biology, Columbia University, New York, NY, USA

2. Department of Biological Sciences, Bridgewater State University, Bridgewater, MA, USA

3. Allen School for Global Health, Washington State University, Pullman, WA, USA

4. Global Vaccines Medical Affairs, Pfizer, Inc., Cambridge, MA, USA

5. Medical Enablement and Quality, Pfizer, Inc., Collegeville, PA, USA

6. Global Vaccines Medical Affairs, Pfizer, Inc., New York, NY, USA

7. United States Medical Affairs, Pfizer, Inc. Collegeville, PA, USA

Corresponding author: Wen Fu, [wf2317@columbia.edu](mailto:wf2317@columbia.edu)

## **Additional file 2: Sampling location, tick data and infection prevalence**


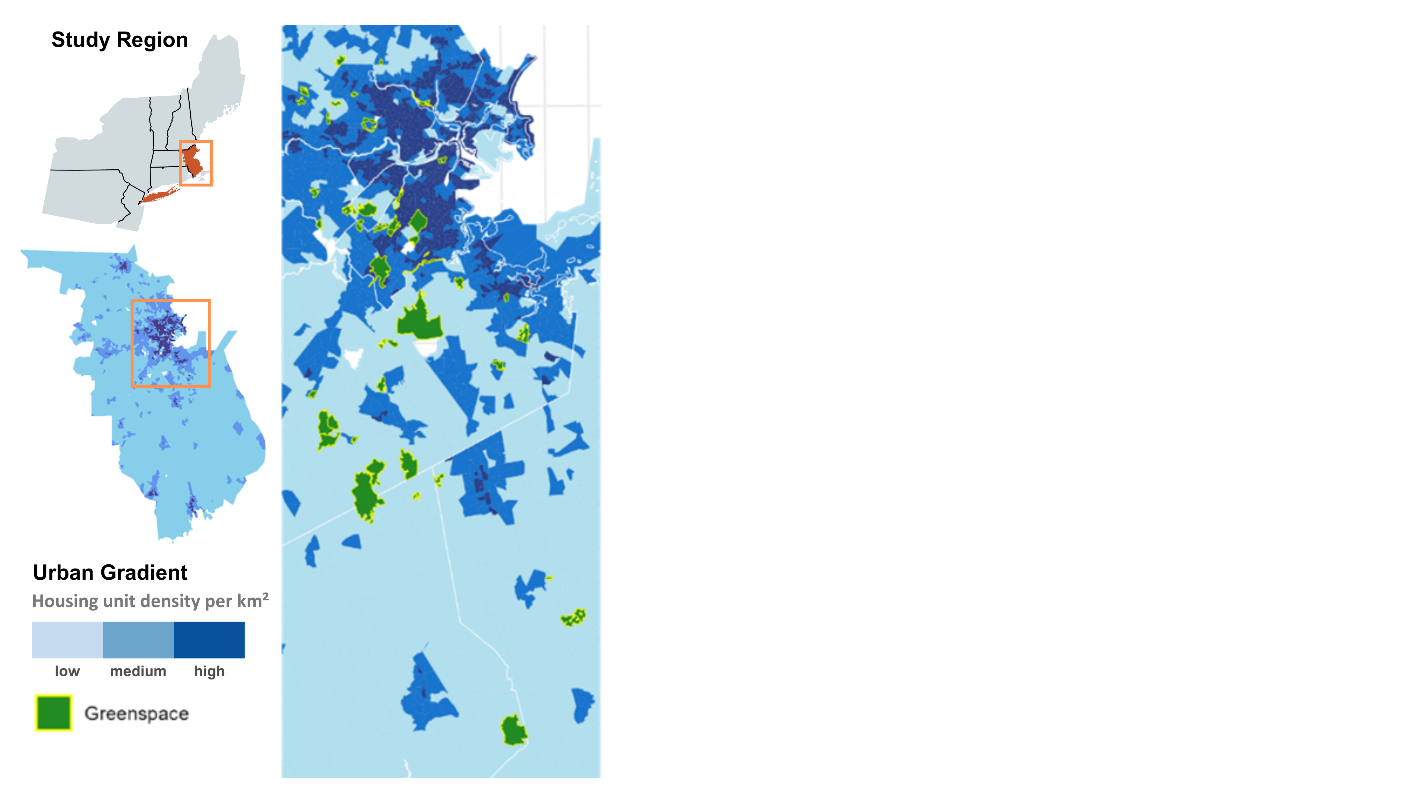
**Fig.S1**. Distribution of the selected 43 greenspaces across a gradient of urban intensity in Greater Boston areas, 2024.

**
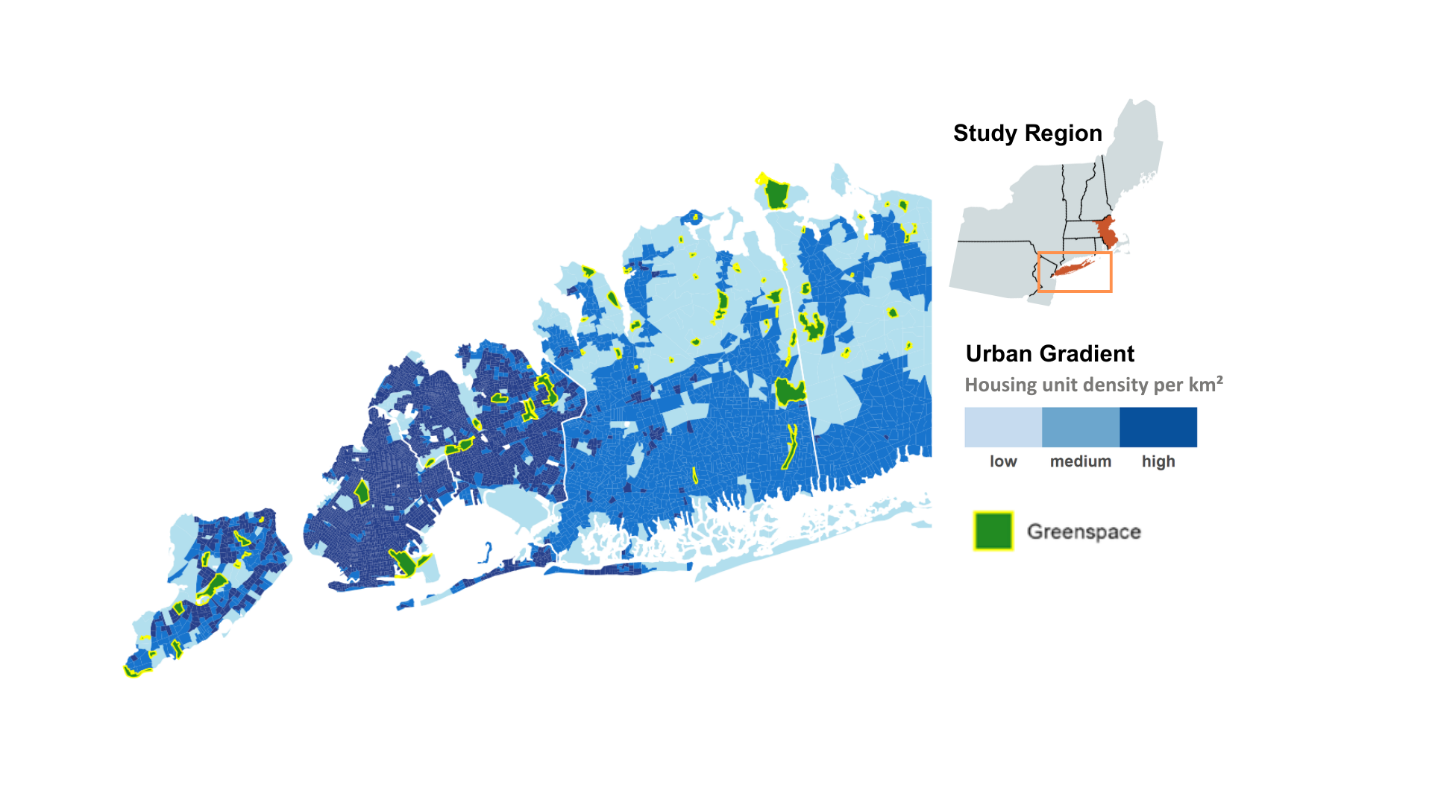
**

**Fig.S2.** Distribution of the selected 49 greenspaces across a gradient of urban intensity in New York City-Long Island, 2024.

**Table S1.** Sampling effort during June–July 2023 to assess Lyme disease hazard across the urbanization gradient in New York City and Long Island, USA.

| **Greenspace** | **County** | **Latitude** | **Longitude** | | **No. nymphs collected** | **Nymphal Infection Prevalence** |
| --- | --- | --- | --- | --- | --- | --- |
| North Forty Natural Area / Floyd Bennett Field | Kings | 40.59913 | -73.8975 | 7 | | 0.14 |
| Marine Park | Kings | 40.59596 | -73.9229 | 0 | | NA |
| Prospect Park | Kings | 40.65692 | -73.9717 | 3 | | 0 |
| Brookville Nature Park | Nassau | 40.81132 | -73.5471 | 236 | | 0.4 |
| Old Westbury Gardens | Nassau | 40.77828 | -73.5978 | 91 | | 0.38 |
| Muttontown Preserve | Nassau | 40.82668 | -73.5367 | 947 | | 0.34 |
| Manetto Hills Park | Nassau | 40.79620 | -73.4511 | 159 | | 0.33 |
| Hope G. Iselin Preserve | Nassau | 40.85277 | -73.5676 | 125 | | 0.3 |
| Tiffany Creek Preserve | Nassau | 40.86228 | -73.5183 | 236 | | 0.3 |
| Humes Preserve | Nassau | 40.87372 | -73.5705 | 83 | | 0.26 |
| Harrison Williams Woods | Nassau | 40.90797 | -73.566 | 4 | | 0.25 |
| Hempstead Natural Area | Nassau | 40.83462 | -73.6673 | 28 | | 0.24 |
| Stillwell Woods Park | Nassau | 40.83722 | -73.4658 | 139 | | 0.24 |
| Suny Old Westbury | Nassau | 40.79468 | -73.5686 | 187 | | 0.24 |
| Trailview State Park 1 | Nassau | 40.81979 | -73.4705 | 38 | | 0.16 |
| Christopher Morley Park | Nassau | 40.78715 | -73.6624 | 14 | | 0.14 |
| William Cullen Bryant Preserve | Nassau | 40.80996 | -73.6404 | 44 | | 0.14 |
| Bailey Arboretum | Nassau | 40.88754 | -73.5839 | 62 | | 0.14 |
| Trailview State Park 2 | Nassau | 40.77735 | -73.4566 | 8 | | 0.13 |
| Sands Point Preserve | Nassau | 40.85989 | -73.6962 | 23 | | 0.13 |
| Fox Hollow Preserve | Nassau | 40.84864 | -73.4853 | 163 | | 0.12 |
| Syosset-Woodbury | Nassau | 40.80808 | -73.4833 | 0 | | NA |
| Roosevelt Preserve | Nassau | 40.67253 | -73.572 | 0 | | NA |
| Clark Botanical Garden | Nassau | 40.77231 | -73.6398 | 0 | | NA |
| Bethpage State Park | Nassau | 40.74789 | -73.4569 | 1 | | 0 |
| Massapequa Preserve | Nassau | 40.69760 | -73.4574 | 2 | | 0 |
| Garvies Point Preserve | Nassau | 40.86075 | -73.6493 | 2 | | 0 |
| Whitney Pond Park | Nassau | 40.78544 | -73.7024 | 5 | | 0 |
| Leeds Pond Preserve | Nassau | 40.81644 | -73.701 | 8 | | 0 |
| Cunningham Park 2 | Queens | 40.74143 | -73.7656 | 1 | | 1 |
| Forest Park 1 | Queens | 40.70383 | -73.8507 | 1 | | 1 |
| Cunningham Park | Queens | 40.72831 | -73.7662 | 0 | | NA |
| Flushing Meadows Park | Queens | 40.72217 | -73.8306 | 0 | | NA |
| John Golden Park | Queens | 40.77399 | -73.7683 | 0 | | NA |
| Forest Park 2 | Queens | 40.70791 | -73.8399 | 0 | | NA |
| Kissena Park | Queens | 40.74522 | -73.8079 | 1 | | 0 |
| Highland Park | Queens | 40.68715 | -73.8842 | 2 | | 0 |
| Alley Pond Park | Queens | 40.74559 | -73.737 | 3 | | 0 |
| North Alley Pond Park | Queens | 40.75915 | -73.7449 | 3 | | 0 |
| Horatio Playground | Queens | 40.759 | -73.7544 | 5 | | 0 |
| Fairview Park | Staten Island | 40.53228 | -74.2314 | 12 | | 0.5 |
| Wolfe's Pond Park | Staten Island | 40.5224 | -74.1937 | 22 | | 0.47 |
| Latourette Park | Staten Island | 40.58235 | -74.1421 | 176 | | 0.42 |
| Conference House Park | Staten Island | 40.49875 | -74.2475 | 18 | | 0.33 |
| Clove Lakes | Staten Island | 40.61914 | -74.115 | 60 | | 0.3 |
| Mount Loretto State Forest | Staten Island | 40.51630 | -74.2227 | 36 | | 0.19 |
| Arden Heights Woods | Staten Island | 40.55801 | -74.1904 | 11 | | 0.18 |
| Deere Park | Staten Island | 40.60685 | -74.1045 | 54 | | 0.16 |
| Todt Hill | Staten Island | 40.59877 | -74.1174 | 15 | | 0.13 |
| Willowbrook Park | Staten Island | 40.60094 | -74.1581 | 16 | | 0.06 |
| Jones Woods | Staten Island | 40.63703 | -74.0904 | 0 | | NA |
| Indian Island County Park | Suffolk | 40.92629 | -72.6299 | 102 | | 0.52 |
| Breezy Park | Suffolk | 40.83893 | -73.435 | 10 | | 0.38 |
| Sunken Meadow State Park | Suffolk | 40.90307 | -73.2604 | 483 | | 0.38 |
| Laurel Ridge Setauket Woods Nature Preserve | Suffolk | 40.91097 | -73.0867 | 19 | | 0.37 |
| Vanderbilt Museum | Suffolk | 40.90527 | -73.3676 | 95 | | 0.33 |
| Pine Neck Marina Park | Suffolk | 40.84821 | -72.5707 | 29 | | 0.31 |
| Southaven County Park | Suffolk | 40.81946 | -72.899 | 74 | | 0.31 |
| Pine Meadow County Park | Suffolk | 40.85321 | -72.7235 | 115 | | 0.31 |
| Lakeland County Park | Suffolk | 40.80420 | -73.1547 | 99 | | 0.3 |
| Froehlich Farm Preserve | Suffolk | 40.83893 | -73.435 | 164 | | 0.3 |
| Caumsett State Historic Park | Suffolk | 40.93009 | -73.4717 | 344 | | 0.3 |
| West Hills County Park | Suffolk | 40.80698 | -73.4289 | 216 | | 0.29 |
| Henry Ingraham Nature Preserve | Suffolk | 40.91652 | -73.3369 | 106 | | 0.26 |
| Dr. Jeffrey Wenig Memorial Park | Suffolk | 40.78177 | -73.3885 | 4 | | 0.25 |
| Connetquot State Park | Suffolk | 40.77314 | -73.1507 | 147 | | 0.25 |
| Millers Pond County Park | Suffolk | 40.84808 | -73.1925 | 46 | | 0.24 |
| Hoyt Farm Nature Preserve | Suffolk | 40.82227 | -73.2705 | 515 | | 0.23 |
| Dix Hills Park | Suffolk | 40.81847 | -73.3322 | 89 | | 0.22 |
| Hidden Pond Park | Suffolk | 40.81949 | -73.1677 | 111 | | 0.22 |
| Sweer Briar Nature Center | Suffolk | 40.86502 | -73.2051 | 75 | | 0.21 |
| Meadowlark Park | Suffolk | 40.89356 | -73.3041 | 413 | | 0.21 |
| Makamah Nature Preserve | Suffolk | 40.91391 | -73.3158 | 380 | | 0.2 |
| Cold Spring Harbor | Suffolk | 40.86298 | -73.4601 | 16 | | 0.19 |
| Schuyler County Park | Suffolk | 40.80982 | -72.9062 | 37 | | 0.19 |
| Robert Cushman Murphy County Park | Suffolk | 40.88388 | -72.8085 | 47 | | 0.19 |
| Prosser Pines Nature Preserve | Suffolk | 40.87145 | -72.9329 | 89 | | 0.18 |
| Good Ground Park | Suffolk | 40.88114 | -72.5276 | 57 | | 0.16 |
| Wildwood State Park | Suffolk | 40.96094 | -72.7951 | 87 | | 0.16 |
| Forsythe Meadow County Park | Suffolk | 40.91845 | -73.142 | 53 | | 0.15 |
| Hedges Creek County Park | Suffolk | 40.76004 | -72.9601 | 60 | | 0.15 |
| Veterans Park | Suffolk | 40.88604 | -73.312 | 188 | | 0.15 |
| Cordwood Landing County Park | Suffolk | 40.96246 | -73.0035 | 84 | | 0.14 |
| Gardiner Farm Park | Suffolk | 40.85270 | -73.3794 | 41 | | 0.13 |
| Rocky Point State Pine Barrens Preserve | Suffolk | 40.92001 | -72.9316 | 53 | | 0.13 |
| Blydenburgh County Park | Suffolk | 40.83722 | -73.2216 | 381 | | 0.13 |
| Bayard Cutting Arboretum | Suffolk | 40.74744 | -73.1632 | 245 | | 0.12 |
| Coindre Hall Park | Suffolk | 40.89488 | -73.4367 | 18 | | 0.11 |
| Farmingville Hills County Park | Suffolk | 40.83926 | -73.0437 | 28 | | 0.11 |
| Glacier Ridge Bike Trail | Suffolk | 40.84727 | -73.0155 | 75 | | 0.07 |
| Gardiner County Park | Suffolk | 40.69642 | -73.274 | 31 | | 0.06 |
| Phragmites Park | Suffolk | 40.88732 | -73.3613 | 25 | | 0.04 |
| Holtsville Park | Suffolk | 40.79854 | -73.0346 | 1 | | 0 |
| Otsego Park | Suffolk | 40.78705 | -73.3136 | 3 | | 0 |

Note: Longitude and latitude represent the estimated centroid of each greenspace and do not correspond to the precise GPS locations of the sampling transects. In 2023, mean infection prevalence was 25.87% in NYC (Staten Island, Kings and Queens) greenspaces and 22.47% in Long Island (Nassau and Suffolk) greenspaces.

**Table S2.** Sampling effort in June and July of 2024 for studying Lyme disease hazard across an urban gradient in Greater Boston, Massachusetts, USA.

| **Greenspace** | **County** | **Latitude** | **Longitude** | **No. nymphs collected** | **Nymphal Infection Prevalence** |
| --- | --- | --- | --- | --- | --- |
| Arnold Arboretum | Suffolk | 42.29866 | -71.1242 | 2 | 0 |
| Allandale Woods | Suffolk | 42.29779 | -71.1387 | 2 | 0 |
| Museum of American Bird Art | Norfolk | 42.16471 | -71.1375 | 59 | 0.27 |
| Beaver Brook | Middlesex | 42.39026 | -71.1972 | 0 | NA |
| Blue Hills Reservation | Norfolk | 42.21761 | -71.094 | 79 | 0.185 |
| Boston Nature Center | Suffolk | 42.28742 | -71.0997 | 4 | 0 |
| Boston Common | Suffolk | 42.35474 | -71.0672 | 0 | NA |
| Brooks Parkhurst Town Forest | Middlesex | 42.43776 | -71.1366 | 3 | 0.33 |
| Bradley Reservation | Norfolk | 42.19997 | -71.1245 | 3 | 0 |
| Brockton Audubon Preserve | Plymouth | 42.08438 | -71.0729 | 11 | 0.36 |
| Borderland State Park | Norfolk | 42.07557 | -71.1539 | 17 | 0.235 |
| braintree Town Forest | Norfolk | 42.18116 | -71.004 | 24 | 0.17 |
| Bussey Brook Meadow | Suffolk | 42.29822 | -71.1187 | 0 | NA |
| Coldspring Park | Middlesex | 42.32786 | -71.2131 | 2 | 0.5 |
| Cunningham Park | Norfolk | 42.25109 | -71.0485 | 2 | 0 |
| Dane Park | Norfolk | 42.31303 | -71.1529 | 0 | NA |
| D. Blakely Hoar Sanctuary | Norfolk | 42.29744 | -71.1619 | 20 | 0.5 |
| Faxon Park | Norfolk | 42.23814 | -70.9951 | 8 | 0.125 |
| Franklin Park | Suffolk | 42.3028 | -71.095 | 0 | NA |
| Fresh Pond | Middlesex | 42.38266 | -71.1551 | 0 | NA |
| Francis William Bird Park | Norfolk | 42.15712 | -71.2161 | 6 | 0 |
| Governor Ames Estate CR | Bristol | 42.07068 | -71.0986 | 2 | 0 |
| Gladeside | Suffolk | 42.27512 | -71.0861 | 8 | 0 |
| Great River Preserve | Plymouth | 41.96676 | -70.9205 | 74 | 0.135 |
| Habitat Education Center & Wildlife Sanctuary | Middlesex | 42.40297 | -71.1883 | 21 | 0.19 |
| James Falzone Memorial Park | Middlesex | 42.41315 | -71.2244 | 5 | 0 |
| Jamaica Pond & Olmsted Park | Suffolk | 42.3229 | -71.1169 | 0 | NA |
| Lost Pond Kennard Conservation Area | Norfolk | 42.31258 | -71.174 | 15 | 0.13 |
| Massasoit State Park | Bristol | 41.8714 | -70.9901 | 43 | 0.24 |
| Moose Hill Wildlife Sanctuary | Norfolk | 42.12724 | -71.1997 | 40 | 0.175 |
| Massapoag Trail & Brook Conservation Area | Norfolk | 42.1187 | -71.1688 | 13 | 0.07 |
| Menotomy Rocks Park | Middlesex | 42.41071 | -71.1675 | 1 | 0 |
| Mystic River Reservation | Middlesex | 42.40393 | -71.089 | 0 | NA |
| Nahanton Park | Middlesex | 42.30102 | -71.2078 | 6 | 0 |
| Neponset River Reservation | Norfolk | 42.26623 | -71.0905 | 0 | NA |
| Pakeen Farm | Norfolk | 42.19966 | -71.1365 | 6 | 0.625 |
| Pond Meadow Park | Norfolk | 42.20625 | -70.9727 | 41 | 0.21 |
| Stony Brook Reservation | Suffolk | 42.26272 | -71.1386 | 5 | 0 |
| Shady pond Conservation Areas | Middlesex | 42.40472 | -71.228 | 4 | 0 |
| Stoughton Memorial Conservation Land | Plymouth | 42.09717 | -71.1071 | 10 | 0.1 |
| Tuckerwood at Chambers Way Conservation Area | Plymouth | 42.00075 | -70.9483 | 33 | 0.235 |
| Whipple Hill Conservation Area | Middlesex | 42.43931 | -71.1833 | 2 | 0 |

Note: Longitude and latitude represent the estimated centroid of each greenspace and do not correspond to the precise GPS locations of the sampling transects.

**Table S3.** Sampling effort in June and July of 2024 for studying Lyme disease hazard across an urban gradient in New York-Long Island, New York, USA.

| **Greenspace** | **County** | **Latitude** | **Longitude** | **No. nymphs collected** | **Nymphal Infection Prevalence** |
| --- | --- | --- | --- | --- | --- |
| Prospect Park 1 | Kings | 40.66505 | -73.9682 | 0 | NA |
| Prospect Park 2 | Kings | 40.65840 | -73.9712 | 0 | NA |
| Bethpage State Park | Nassau | 40.75362 | -73.4637 | 3 | 0 |
| Brookville Nature Park | Nassau | 40.81060 | -73.5471 | 193 | 0.36 |
| Christopher Morley Park | Nassau | 40.78728 | -73.6632 | 0 | NA |
| Clark Botanic Gardens | Nassau | 40.77223 | -73.6393 | 0 | NA |
| Fox Hollow Preserve | Nassau | 40.84814 | -73.4858 | 18 | 0.11 |
| Hope Goddard Iselin Preserve | Nassau | 40.85427 | -73.5677 | 45 | 0.22 |
| Manetto Hills Park | Nassau | 40.79027 | -73.4543 | 75 | 0.22 |
| Massapequa County Park | Nassau | 40.69995 | -73.4536 | 3 | 0 |
| Muttontown Preserve | Nassau | 40.83687 | -73.5339 | 76 | 0.24 |
| Old Westbury Gardens | Nassau | 40.77529 | -73.5929 | 37 | 0.305 |
| SUNY Old Westbury | Nassau | 40.79512 | -73.5708 | 37 | 0.135 |
| Stillwell Woods County Park | Nassau | 40.83458 | -73.4717 | 54 | 0.31 |
| Tiffany Creek Preserve | Nassau | 40.85910 | -73.5166 | 69 | 0.32 |
| Trail View State Park 1 | Nassau | 40.81778 | -73.4709 | 17 | 0.07 |
| Trail View State Park 2 | Nassau | 40.77907 | -73.4561 | 6 | 0.2 |
| Whitney Pond Park | Nassau | 40.78543 | -73.7028 | 5 | 0 |
| William Cullen Bryant Preserve | Nassau | 40.80829 | -73.6438 | 26 | 0.27 |
| Alley Pond Park | Queens | 40.74379 | -73.7411 | 2 | 0.5 |
| Cunningham Park 1 | Queens | 40.72929 | -73.7684 | 0 | NA |
| Cunningham Park 2 | Queens | 40.74210 | -73.7669 | 0 | NA |
| Flushing Meadows Corona Park | Queens | 40.72285 | -73.8304 | 0 | NA |
| Forest Park 1 | Queens | 40.70366 | -73.8511 | 0 | NA |
| Forest Park 2 | Queens | 40.70659 | -73.8398 | 1 | 0 |
| Highland Park | Queens | 40.68705 | -73.8875 | 2 | 0 |
| Horatio Playground | Queens | 40.75920 | -73.7541 | 16 | 0.125 |
| Kissena Park | Queens | 40.74371 | -73.8077 | 1 | 0 |
| North Alley Pond Park | Queens | 40.76269 | -73.7467 | 0 | NA |
| Arden Heights Woods | Staten Island | 40.55871 | -74.1814 | 2 | 0.5 |
| Blue Heron Park | Staten Island | 40.53076 | -74.177 | 4 | 0.6 |
| Clove Lakes Park | Staten Island | 40.61786 | -74.113 | 4 | 0 |
| Conference House Park | Staten Island | 40.50119 | -74.2516 | 16 | 0.43 |
| Deere Park | Staten Island | 40.60654 | -74.1053 | 7 | 0.22 |
| Fairview Park | Staten Island | 40.53201 | -74.2317 | 14 | 0.36 |
| Jones Woods Park | Staten Island | 40.63676 | -74.0911 | 0 | NA |
| Latourette Park | Staten Island | 40.57902 | -74.1458 | 52 | 0.12 |
| Lemon Creek Park | Staten Island | 40.52207 | -74.203 | 14 | 0.14 |
| Long Pond Park | Staten Island | 40.51432 | -74.2301 | 36 | 0.13 |
| Mount Loretto Unique Area | Staten Island | 40.50681 | -74.2181 | 7 | 0.29 |
| North Mount Loretto State Forest | Staten Island | 40.51726 | -74.2243 | 18 | 0.08 |
| Willowbrook Park | Staten Island | 40.60156 | -74.1567 | 3 | 0 |
| Wolfe's Pond Park | Staten Island | 40.51850 | -74.1907 | 10 | 0.6 |
| Cold Spring Harbor | Suffolk | 40.86597 | -73.4603 | 17 | 0.18 |
| Dix Hills Park | Suffolk | 40.82069 | -73.3316 | 2 | 1 |
| Froehlich Farm Preserve | Suffolk | 40.84172 | -73.4351 | 61 | 0.25 |
| Gardiner County Park | Suffolk | 40.85269 | -73.3795 | 11 | 0 |
| Veteran's / Knolls | Suffolk | 40.88524 | -73.309 | 43 | 0.26 |
| West Hills County Park | Suffolk | 40.80231 | -73.4195 | 73 | 0.145 |

Note: Longitude and latitude represent the estimated centroid of each greenspace and do not correspond to the precise GPS locations of the sampling transects.
