# Supplementary material for: Mapping tick-borne hazard across gradients of urban intensity in metropolitan regions
Source: Parasit Vectors. 2026 May 25;19:295. doi: 10.1186/s13071-026-07448-4 (PMC13386969; doi:10.1186/s13071-026-07448-4)
Supplement: Supplementary file 3 — Supplementary Material 3. [file 13071_2026_7448_MOESM3_ESM.docx]

**Mapping tick-borne hazard across gradients of urban intensity in metropolitan regions**

Wen Fu^1*^, Marie V. Lilly^1^, Sung-Joo Lee^1^, Heather Kopsco^1^, Thilina Surasinghe^2^, Maria Del Pilar Fernandez^3^, Viorel Popescu^1^, James Stark^4^, Juanita Edwards^5^, L. Hannah Gould^6^, Patrick H. Kelly^7^, Maria A. Diuk-Wasser^1^

1. Department of Ecology, Evolution, and Environmental Biology, Columbia University, New York, NY, USA

2. Department of Biological Sciences, Bridgewater State University, Bridgewater, MA, USA

3. Allen School for Global Health, Washington State University, Pullman, WA, USA

4. Global Vaccines Medical Affairs, Pfizer, Inc., Cambridge, MA, USA

5. Medical Enablement and Quality, Pfizer, Inc., Collegeville, PA, USA

6. Global Vaccines Medical Affairs, Pfizer, Inc., New York, NY, USA

7. United States Medical Affairs, Pfizer, Inc. Collegeville, PA, USA

Corresponding author: Wen Fu, [wf2317@columbia.edu](mailto:wf2317@columbia.edu)

## **Additional file 3: Statistical Model equations and covariance specifications**

We developed three spatial generalized linear mixed effects (GLMM) models (binomial and negative binomial families) to estimate: (1) the probability of nymph presence, (2) the density of questing nymphs (DON), and (3) the density of *B. burgdorferi*–infected nymphs (DIN). Models were trained using 2023 tick surveillance data from 94 NYC-LI greenspaces and evaluated through internal validation with same-year data and external validation using independent 2024 data from NYC–LI and Greater Boston. For each model, response variables were defined at the transect level by aggregating observations to the GPS start point (World Geodetic System 1984) of each transect. Accordingly, each 100-m grid cell in the predictive maps represents the expected outcome of a randomly placed transect within that area.

We used a logistic regression model with spatial random effects to predict the probability of questing nymph presence (equation 1) and two separate negative binomial models to estimate DON and DIN (equation 2).

$Logit(P\left( Y_{\left( s \right)}=1 \right))=X_{1}\left( s \right)^{\top}\beta_{1}+w_{1}\left( s \right)$ (1)

$log (\mu\left( s \right))=X_{2}\left( s \right)^{\top}\beta_{2}+w_{2}\left( s \right)$ (2)

Where $Y_{(s)}$ denote the tick presence with expected numbers $\mu\left( s \right)$ at transect $s\in S,$ where *S* is the study region. $X_{1}\left( s \right)$, $X_{2}\left( s \right)$are the vectors of covariates hypothesized to be associated with tick presence and densities (DON and DIN), respectively. $\beta_{1}$**,** $\beta_{2}$ represent the respective regression coefficients. All spatial GLMMs incorporated a spatial Gaussian process component to account for residual spatial autocorrelation and unmeasured ecological variation [1]. We included spatially structured random effects $w_{1}\left( s \right)$and $w_{2}\left( s \right),$modeled as a zero-mean Gaussian process with Matérn covariance function $C_{1}$_,_ denote as $w_{1}\left( s \right), w_{2}\left( s \right)\sim GP\left( 0,C_{1}\left( \cdot,\cdot\right) \right)$.

## **Model selection and validation**

We fit global spatial models for each outcome: tick presence, DON, and DIN, using 2023 NYC–LI data. Optimal model structures were identified using a drop-one covariate selection procedure, in which each predictor was sequentially removed from the global model and model fit was assessed using penalized likelihood ratio tests that accounted for spatial correlation [1,2]. Covariates with p-values > 0.05 were excluded, and reduced models were compared with global models using conditional Akaike information criterion (cAIC), with the lowest cAIC selected as the final model for spatial prediction. To assess potential effect modification, we additionally evaluated model specifications including an interaction between percent impervious surface and functional connectivity. Interaction terms were retained only if statistically significant and if they improved model performance, as indicated by a lower cAIC.

The robustness of the final presence model was evaluated by comparing predictive discrimination across three presence thresholds (≥1, ≥2, and ≥3 nymphs per transect). Internal validation was conducted using grouped five-fold cross-validation, in which the dataset was randomly partitioned into five folds; models were trained on four folds and evaluated on the remaining fold, rotating through all folds so that each observation was used once for testing. Out-of-fold predictions were used to quantify overall performance.

For external validation, models trained on 2023 NYC–LI data were applied to independent 2024 datasets from NYC–LI and Greater Boston. Predictor variables were standardized using means and standard deviations from the training data to ensure comparability. Model discrimination was assessed using the area under the receiver operating characteristic curve (AUC), and calibration was evaluated using logistic regression of observed outcomes on the logit-transformed predicted probabilities. For count-based models (DON and DIN), model fit was further evaluated using pseudo-R² metrics and residual diagnostics.

**References**

1. Rousset F, Ferdy JB. Testing environmental and genetic effects in the presence of spatial autocorrelation. Ecography. Blackwell Publishing Ltd; 2014;37:781–90. https://doi.org/10.1111/ECOG.00566

2. Alain F. Zuur, Elena N.Ieno, Neil J. Walker, Anatoly A. Saveliev, GrahamM. Smith. Mixed Effects Models and Extensions in Ecology with R. 2008. https://doi.org/10.1007/978-0-387-87458-6
