# Supplementary material for: Mapping tick-borne hazard across gradients of urban intensity in metropolitan regions
Source: Parasit Vectors. 2026 May 25;19:295. doi: 10.1186/s13071-026-07448-4 (PMC13386969; doi:10.1186/s13071-026-07448-4)
Supplement: Supplementary file 4 — Supplementary Material 4. [file 13071_2026_7448_MOESM4_ESM.docx]

**Mapping tick-borne hazard across gradients of urban intensity in metropolitan regions**

Wen Fu^1*^, Marie V. Lilly^1^, Sung-Joo Lee^1^, Heather Kopsco^1^, Thilina Surasinghe^2^, Maria Del Pilar Fernandez^3^, Viorel Popescu^1^, James Stark^4^, Juanita Edwards^5^, L. Hannah Gould^6^, Patrick H. Kelly^7^, Maria A. Diuk-Wasser^1^

1. Department of Ecology, Evolution, and Environmental Biology, Columbia University, New York, NY, USA

2. Department of Biological Sciences, Bridgewater State University, Bridgewater, MA, USA

3. Allen School for Global Health, Washington State University, Pullman, WA, USA

4. Global Vaccines Medical Affairs, Pfizer, Inc., Cambridge, MA, USA

5. Medical Enablement and Quality, Pfizer, Inc., Collegeville, PA, USA

6. Global Vaccines Medical Affairs, Pfizer, Inc., New York, NY, USA

7. United States Medical Affairs, Pfizer, Inc. Collegeville, PA, USA

Corresponding author: Wen Fu, [wf2317@columbia.edu](mailto:wf2317@columbia.edu)

**Additional file 4: Predictor and buffer size selection**

**Table S4**. Selection of Optimal buffer sizes for landscape composition and configuration predictors based on conditional AIC in Modeling the presence of *Ixodes scapularis* nymphs in New York City-Long Island, NY, USA.

| Covariate | Coefficient estimate | *t* value | Buffer size, m | Conditional AIC |
| --- | --- | --- | --- | --- |
| Tree Canopy |  |  |  |  |
|  | **0.674** | **7.045** | **100** | **1132.481** |
|  | 0.701 | 5.274 | 500 | 1160.434 |
|  | 0.966 | 5.787 | 1,000 | 1155.524 |
| Connectivity |  |  |  |  |
|  | 0.461 | 3.250 | 100 | 1164.972 |
|  | 0.611 | 3.784 | 500 | 1163.974 |
|  | **0.637** | **3.684** | **1,000** | **1162.161** |
| Impervious surface |  |  |  |  |
|  | -0.530 | -5.405 | 100 | 1151.247 |
|  | -0.765 | -5.468 | 500 | 1155.069 |
|  | **-1.132** | **-6.093** | **1,000** | **1150.325** |
| Soil sand |  |  |  |  |
|  | 0.414 | 3.742 | 100 | 1158.892 |
|  | 0.616 | 4.610 | 500 | 1158.900 |
|  | **0.754** | **4.988** | **1,000** | **1156.063** |
| Evergreen Forest |  |  |  |  |
|  | 0.125 | 0.836 | 100 | 1166.388 |
|  | -0.136 | -0.787 | 500 | 1165.532 |
|  | -0.126 | -0.664 | 1,000 | 1165.604 |
| Decidious Forest |  |  |  |  |
|  | **0.600** | **5.533** | **100** | **1151.323** |
|  | 0.578 | 4.235 | 500 | 1158.611 |
|  | 0.663 | 4.420 | 1,000 | 1157.788 |
| Grassland |  |  |  |  |
|  | **-0.342** | **-2.425** | **100** | **1161.540** |
|  | 0.025 | 0.157 | 500 | 1166.710 |
|  | -0.115 | -0.582 | 1,000 | 1166.188 |
| Woody wetlands |  |  |  |  |
|  | 0.144 | 1.162 | 100 | 1165.631 |
|  | 0.098 | 0.562 | 500 | 1166.767 |
|  | 0.154 | 0.833 | 1,000 | 1166.424 |
| Open waters |  |  |  |  |
|  | -0.035 | -0.309 | 100 | 1167.577 |
|  | **0.132** | **0.926** | **500** | **1165.151** |
|  | 0.033 | 0.221 | 1,000 | 1166.471 |

Each predictor was extracted at 100 m, 500 m, and 1,000 m buffers. The optimal buffer size for each covariate is indicated in bold, selected based on the lowest conditional AIC (cAIC) value that differs by at least 2 units from other buffer sizes. When cAIC differences were less than 2 units, no single buffer size was considered definitively optimal. In such cases, buffer sizes with comparable cAIC values may be used flexibly in model selection, while considering potential collinearity with other predictors at the same spatial scale.

**Table S5**. Univariate models with fixed-scale weather-related predictors and landscape metrics for Ixodes scapularis nymph presence in New York, NY, USA.

| Covariate | Coefficient estimate | *t* value | Conditional AIC |
| --- | --- | --- | --- |
| Air temperature* | -0.56 | -2.21 | 1166.514 |
| Vapor Pressure Deficit | -0.38 | -1.43 | 1165.281 |
| Wildland urban interface (categorical) |  |  | 1166.065 |
| Reference (non-WUI) |  | - |  |
| Class 2 (interface) | 0.40 | 0.88 |  |
| Class 3 (intermix) | -0.91 | -0.87 |  |

Air temperature was the only statistically significant fixed-scale predictor, showing a negative association with tick presence (Estimate = –0.56, *t* = –2.21). The temperature variable represents the average daily mean from June to July during the tick sampling season, suggesting that higher temperatures may reduce the likelihood of detecting questing nymphs. Vapor pressure deficit and wildland–urban interface (WUI) classifications were not statistically significant predictors.

## **Binomial model for predicting the presence of *Ixodes* *scapularis* nymphs**

**Table S6.** Full (global) binomial model predicting tick presence without interaction terms.

| Covariate | Buffer (m) | Estimate | Conditional SE* | *t*-value | *p*-value |
| --- | --- | --- | --- | --- | --- |
| Evergreen Forest | 100 | -0.04 | 0.15 | -0.29 | 0.77 |
| Woody Wetlands | 500 | -0.06 | 0.16 | -0.38 | 0.70 |
| Deciduous Forest | 100 | -0.001 | 0.16 | -0.005 | 0.99 |
| Open Water | 500 | 0.09 | 0.14 | 0.63 | 0.53 |
| Grassland | 100 | -0.07 | 0.16 | -0.21 | 0.83 |
| Tree Canopy Cover | 100 | 0.61 | 0.14 | 4.42 | <0.001 |
| Soil sand | 1,000 | 0.04 | 0.27 | 0.15 | 0.88 |
| Air Temperature | – | -0.16 | 0.29 | -0.56 | 0.57 |
| Vapor Pressure Deficit | – | -0.15 | 0.26 | -0.59 | 0.56 |
| WUI (class 2) | – | 0.26 | 0.41 | 0.62 | 0.53 |
| WUI (class 3) | – | -0.48 | 0.91 | -0.52 | 0.60 |
| Connectivity (Medium) | 1,000 | 0.33 | 0.32 | 1.03 | 0.30 |
| Connectivity (High) | 1,000 | 0.02 | 0.59 | 0.03 | 0.98 |
| Impervious Surface | 1,000 | -0.77 | 0.30 | -2.61 | 0.009 |

*Conditional SE = Standard error of the fixed effect estimate, accounting for uncertainty given the estimated spatial random effect structure.

**Table S7**. Full (global) binomial model predicting tick presence with interaction between connectivity class and impervious surfaces.

| Covariate | Buffer (m) | Estimate | Conditional SE* | *t*-value | *p*-value |
| --- | --- | --- | --- | --- | --- |
| Intercept |  | 0.63 | 0.46 | 1.37 | 0.17 |
| Evergreen Forest | 100 | -0.03 | 0.15 | -0.18 | 0.86 |
| Woody Wetlands | 500 | -0.05 | 0.16 | -0.29 | 0.77 |
| Deciduous Forest | 100 | -0.001 | 0.16 | -0.006 | 0.99 |
| Open Water | 500 | 0.07 | 0.14 | 0.53 | 0.60 |
| Grassland | 100 | -0.05 | 0.15 | -0.33 | 0.75 |
| Tree Canopy Cover | 100 | 0.60 | 0.14 | 4.37 | <0.001 |
| Soil sand | 1,000 | 0.08 | 0.27 | 0.29 | 0.77 |
| Air temperature | – | -0.14 | 0.29 | -0.49 | 0.62 |
| Vapor pressure deficit | – | -0.17 | 0.26 | -0.66 | 0.51 |
| WUI (class 2) | – | 0.23 | 0.41 | 0.55 | 0.58 |
| WUI (class 3) | – | -0.53 | 0.92 | -0.57 | 0.57 |
| Connectivity (Medium) | 1,000 | 0.35 | 0.32 | 1.08 | 0.28 |
| Connectivity (High) | 1,000 | 0.30 | 1.13 | 0.26 | 0.79 |
| Impervious Surface | 1,000 | -0.85 | 0.31 | -2.73 | 0.006 |
| Connectivity (Medium) x Impervious | 1,000 | 0.30 | 0.39 | 0.77 | 0.44 |
| Connectivity (High) x Impervious | 1,000 | 0.43 | 0.94 | 0.46 | 0.64 |

*Conditional SE = Standard error of the fixed effect estimate, accounting for uncertainty given the estimated spatial random effect structure.

**Table S8.** Likelihood-ratio tests for single-term deletions from the full model.

| Covariate | Buffer (m) | Chi-squared | df | *p*-value |
| --- | --- | --- | --- | --- |
| Evergreen Forest | 100 | 0.0772 | 1 | 0.78 |
| Woody Wetlands | 500 | 0.1347 | 1 | 0.71 |
| Deciduous Forest | 100 | 0.0000 | 1 | 0.99 |
| Open Water | 500 | 0.3597 | 1 | 0.55 |
| Grassland | 100 | 0.0450 | 1 | 0.83 |
| Tree Canopy Cover | 100 | 19.0852 | 1 | <0.001 |
| Soil sand | 1,000 | 0.0035 | 1 | 0.89 |
| Air Temperature | – | 0.2803 | 1 | 0.60 |
| Vapor pressure deficit | – | 0.3026 | 1 | 0.57 |
| Wildland-Urban Interface | – | 0.6176 | 1 | 0.73 |
| Connectivity | 1,000 | 1.2444 | 1 | 0.54 |
| Impervious Surface | 1,000 | 5.8036 | 1 | 0.015 |

**Table S9.** Model selection results for binomial models ranked by conditional AIC (cAIC).

| Model Description | cAIC | Rank |
| --- | --- | --- |
| Reduced model without an interaction term | 1121.75 | 1 |
| Reduced model with an interaction term | 1126.83 | 2 |
| Full model without interaction | 1133.23 | 3 |
| Full model with interaction | 1135.68 | 4 |

**Table S10**. Full (global) negative binomial model for predicting *Ixodes scapularis* nymph density (without an interaction term).

| Covariate | Buffer (m) | Estimate | Conditional SE | t-value | p-value |
| --- | --- | --- | --- | --- | --- |
| Evergreen Forest | 100 | -0.10 | 0.09 | -1.11 | 0.27 |
| Woody Wetlands | 500 | -0.20 | 0.11 | -1.75 | 0.08 |
| Deciduous Forest | 100 | 0.04 | 0.10 | 0.43 | 0.67 |
| Open Water | 500 | 0.09 | 0.09 | 0.35 | 0.72 |
| Grassland | 100 | 0.04 | 0.11 | 0.38 | 0.74 |
| Tree Canopy Cover | 100 | 0.38 | 0.09 | 4.38 | <0.001 |
| Soil sand | 1,000 | -0.19 | 0.21 | -0.90 | 0.37 |
| Air Temperature | 1,000 | -0.13 | 0.21 | -0.62 | 0.53 |
| Vapor pressure deficit | 1,000 | -0.30 | 0.18 | -1.54 | 0.12 |
| WUI (class 2) | – | 0.30 | 0.30 | 0.99 | 0.32 |
| WUI (class 3) | – | -0.31 | 0.79 | -0.39 | 0.69 |
| Connectivity (Med) | 1,000 | 0.20 | 0.25 | 0.66 | 0.51 |
| Connectivity (High) | 1,000 | 0.41 | 0.46 | 0.90 | 0.37 |
| Impervious Surface | 1,000 | -1.07 | 0.24 | -4.48 | <0.001 |

*Conditional SE = Standard error of the fixed effect estimate, accounting for uncertainty given the estimated spatial random effect structure.


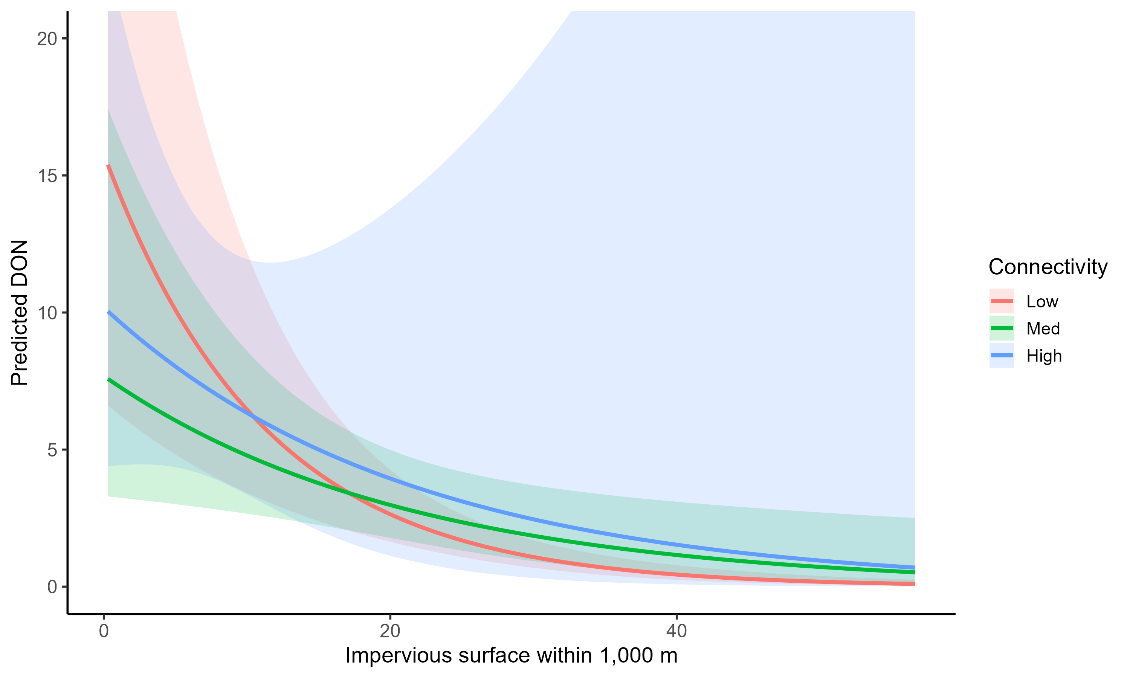


**Fig.S3**. Interaction between percent impervious surface (x-axis) at 1,000 m buffer and functional connectivity class (low, medium, high) on predicted density of *Ixodes scapularis* nymphs (DON/100 m²) at each sampling transect across 94 greenspaces in New York City–Long Island, 2023. Predictions are based on a negative binomial spatial GLMM, with 95% credible intervals. The negative association between impervious surface and DON was attenuated in medium- and high-connectivity landscapes, although uncertainty was larger for the high-connectivity group.

**Table S11**. Full (global) negative binomial model for predicting *Ixodes scapularis* nymph density (with an interaction term).

| Covariate | Buffer (m) | Estimate | Conditional SE | *t*-value | *p*-value |
| --- | --- | --- | --- | --- | --- |
| Intercept |  | 0.50 | 0.24 | 2.11 | 0.035 |
| Evergreen Forest | 100 | -0.19 | 0.09 | -1.09 | 0.28 |
| Woody Wetlands | 500 | -0.22 | 0.11 | -1.94 | 0.05 |
| Deciduous Forest | 100 | 0.04 | 0.10 | 0.40 | 0.69 |
| Open Water | 500 | 0.02 | 0.09 | 0.18 | 0.86 |
| Grassland Herbaceous | 100 | 0.01 | 0.11 | 0.13 | 0.89 |
| Tree Canopy Cover | 100 | 0.38 | 0.09 | 4.38 | <0.001 |
| Soil sand | 1,000 | -0.11 | 0.21 | -0.52 | 0.60 |
| Air temperature | – | -0.10 | 0.21 | -0.45 | 0.65 |
| Vapor pressure deficit | – | -0.30 | 0.18 | -1.71 | 0.09 |
| WUI (class 2) | – | 0.23 | 0.30 | 0.78 | 0.44 |
| WUI (class 3) | – | -0.32 | 0.77 | -0.41 | 0.68 |
| Connectivity (Med) | 1,000 | 0.33 | 0.26 | 1.27 | 0.20 |
| Connectivity (High) | 1,000 | 0.37 | 0.80 | 0.46 | 0.65 |
| Impervious Surface | 1,000 | -1.18 | 0.25 | -4.81 | <0.001 |
| Connectivity (Med) x Impervious | 1,000 | 0.58 | 0.28 | 2.09 | 0.04 |
| Connectivity (High) x Impervious | 1,000 | 0.23 | 0.66 | 0.34 | 0.73 |

*Conditional SE = Standard error of the fixed effect estimate, accounting for uncertainty given the estimated spatial random effect structure.

**Table S12.** Likelihood-ratio tests for single-term deletions from the full negative binomial model.

| Covariate | Buffer (m) | Chi2 LR | df | *p*-value |
| --- | --- | --- | --- | --- |
| Evergreen Forest | 100 | 1.19 | 1 | 0.28 |
| Woody Wetlands | 500 | 2.88 | 1 | 0.09 |
| Deciduous Forest | 100 | 0.17 | 1 | 0.68 |
| Open Water | 500 | 0.12 | 1 | 0.73 |
| Grassland | 100 | 0.10 | 1 | 0.75 |
| Tree Canopy Cover | 100 | 18.14 | 1 | <0.001 |
| Soil sand | 1,000 | 0.69 | 1 | 0.41 |
| Air temperature | – | 0.38 | 1 | 0.54 |
| Vapor pressure deficit | – | 2.34 | 1 | 0.13 |
| WUI | – | 1.03 | 2 | 0.60 |
| Connectivity | 1,000 | 0.70 | 2 | 0.68 |
| Impervious Surface | 1,000 | 16.43 | 1 | <0.001 |

**Table 13.** Model selection results for negative binomial models predicting *Ixodes scapularis* nymph density, ranked by conditional AIC (cAIC).

| Model Description | cAIC | Rank |
| --- | --- | --- |
| Reduced model with an interaction term | 5330.121 | 1 |
| Full model without interaction | 5332.315 | 2 |
| Full model with interaction | 5334.982 | 3 |
