# Supplementary material for: Mapping tick-borne hazard across gradients of urban intensity in metropolitan regions
Source: Parasit Vectors. 2026 May 25;19:295. doi: 10.1186/s13071-026-07448-4 (PMC13386969; doi:10.1186/s13071-026-07448-4)
Supplement: Supplementary file 5 — Supplementary Material 5. [file 13071_2026_7448_MOESM5_ESM.docx]

**Mapping tick-borne hazard across gradients of urban intensity in metropolitan regions**

Wen Fu^1*^, Marie V. Lilly^1^, Sung-Joo Lee^1^, Heather Kopsco^1^, Thilina Surasinghe^2^, Maria Del Pilar Fernandez^3^, Viorel Popescu^1^, James Stark^4^, Juanita Edwards^5^, L. Hannah Gould^6^, Patrick H. Kelly^7^, Maria A. Diuk-Wasser^1^

1. Department of Ecology, Evolution, and Environmental Biology, Columbia University, New York, NY, USA

2. Department of Biological Sciences, Bridgewater State University, Bridgewater, MA, USA

3. Allen School for Global Health, Washington State University, Pullman, WA, USA

4. Global Vaccines Medical Affairs, Pfizer, Inc., Cambridge, MA, USA

5. Medical Enablement and Quality, Pfizer, Inc., Collegeville, PA, USA

6. Global Vaccines Medical Affairs, Pfizer, Inc., New York, NY, USA

7. United States Medical Affairs, Pfizer, Inc. Collegeville, PA, USA

Corresponding author: Wen Fu, [wf2317@columbia.edu](mailto:wf2317@columbia.edu)

## **Additional file 5: Model diagnostics and validation**


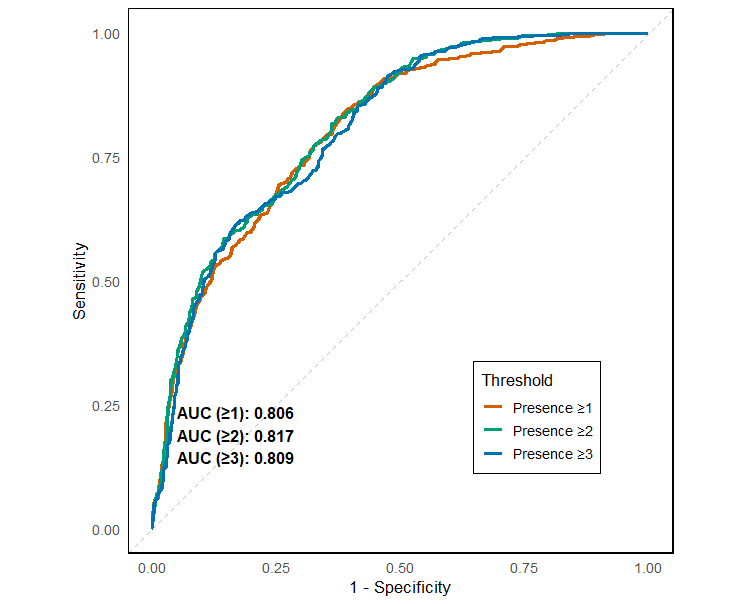
**Fig.S4**. Receiver Operating Characteristic (ROC) curves for logistic regression models predicting nymph presence under three threshold definitions (≥1, ≥2, and ≥3 nymphs per transect), based on 2023 NYC–LI tick data. Area under the curve (AUC) values were 0.806, 0.817, and 0.809, respectively, indicating robust model performance across varying definitions of tick presence.


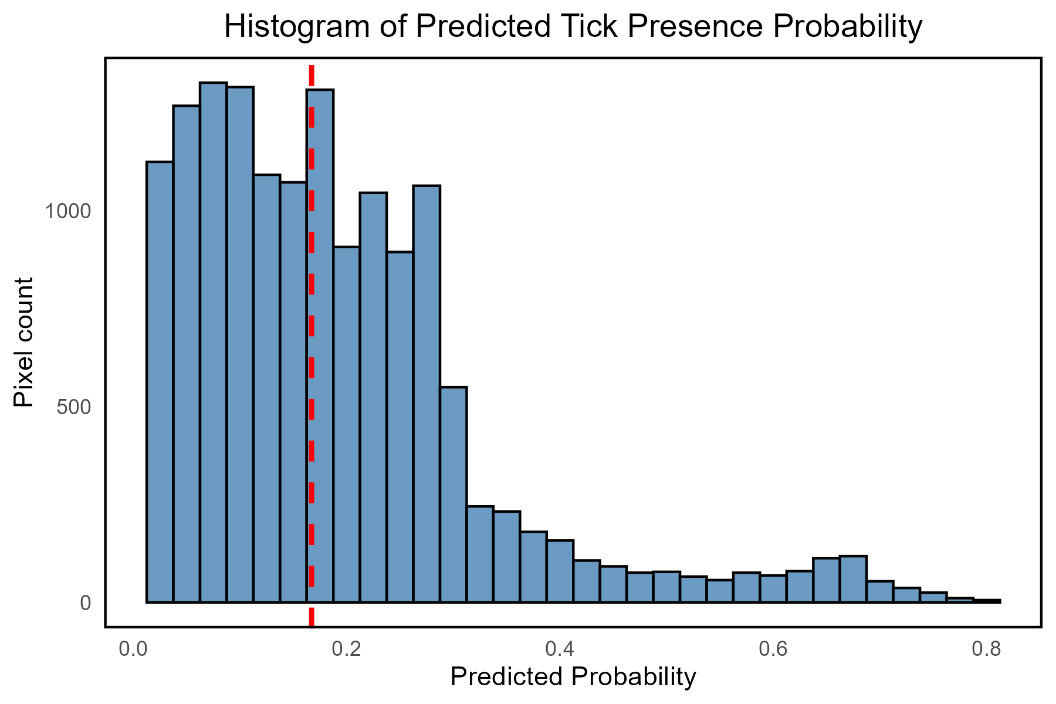


**Fig.S5**. Distribution of predicted probabilities of *Ixodes scapularis* nymph presence across 14,966 100 m prediction units in NYC–LI. Predicted values ranged from 0.01 to 0.80, with a median of 0.17 (red dashed line), interquartile range of 0.08 (Q1) to 0.26 (Q3), and a mean of 0.19, indicating a right-skewed distribution with most values falling below 0.30.


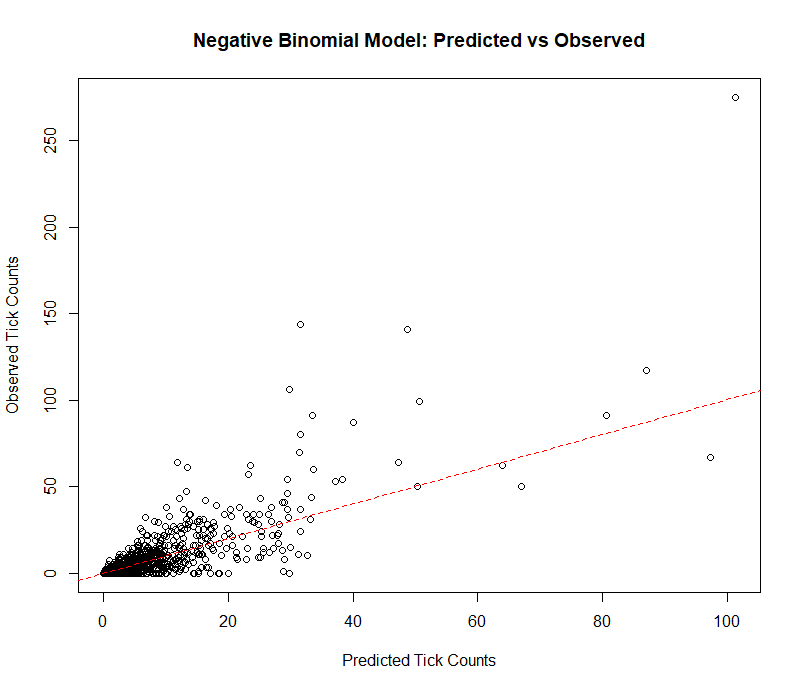
**Fig.S6**. Predicted vs. observed tick counts from the spatial negative binomial model (D0N) in NYC–LI, 2023. Each point represents a transect-level observation comparing predicted and observed Ixodes scapularis nymph counts. The red dashed line indicates the 1:1 reference line. While the model captures overall trends, it tends to underestimate extreme tick abundances, highlighting challenges in modeling overdispersed count data with high variability.

**
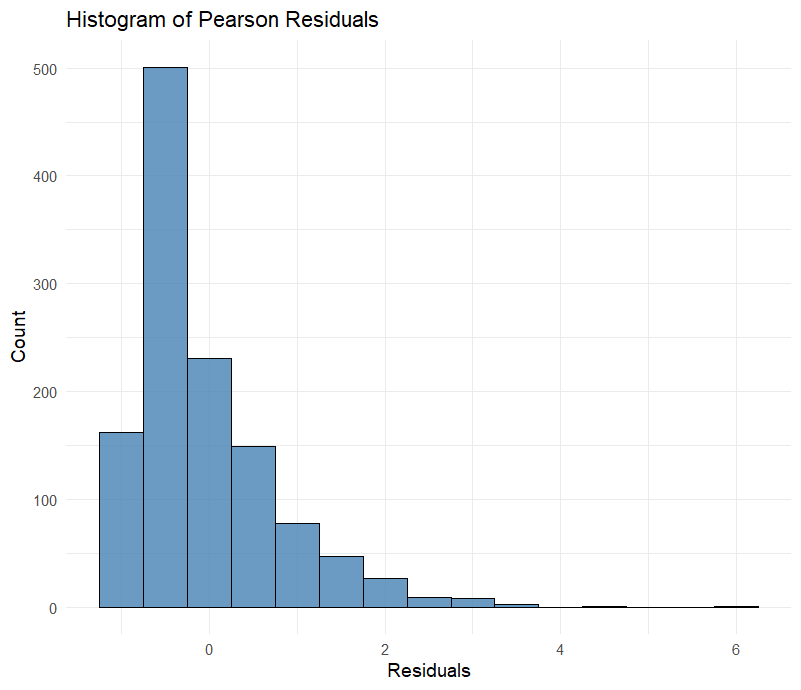
**

**Fig.S7**. Histogram of Pearson residuals from DON model (New York, 2023). The distribution of Pearson residuals is right-skewed, with most residuals concentrated near zero.

## **Model external validation, 2024 NYC-LI**

**
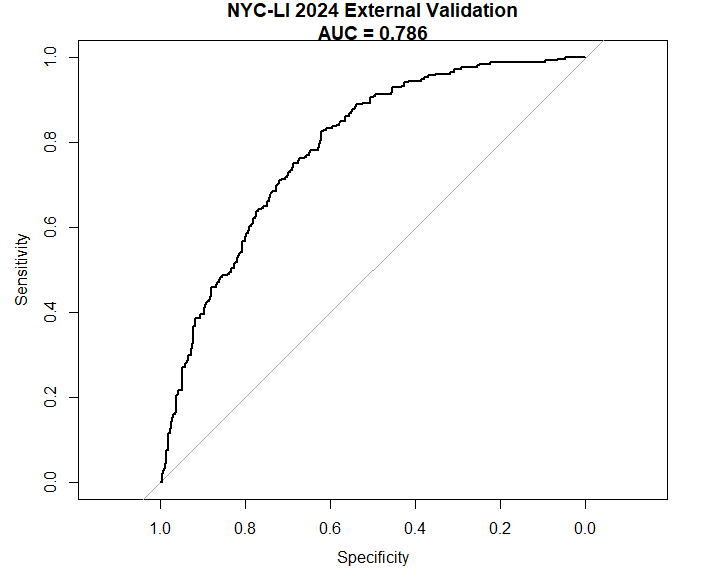
**

**Fig.S8**. Receiver Operating Characteristic (ROC) curve evaluating the external performance of the 2023 NYC–LI logistic model predicting *Ixodes scapularis* nymph presence, validated against 2024 NYC–LI data. The model demonstrated good discrimination, with an AUC of 0.786.

## **Model external validation, 2024 Greater Boston**


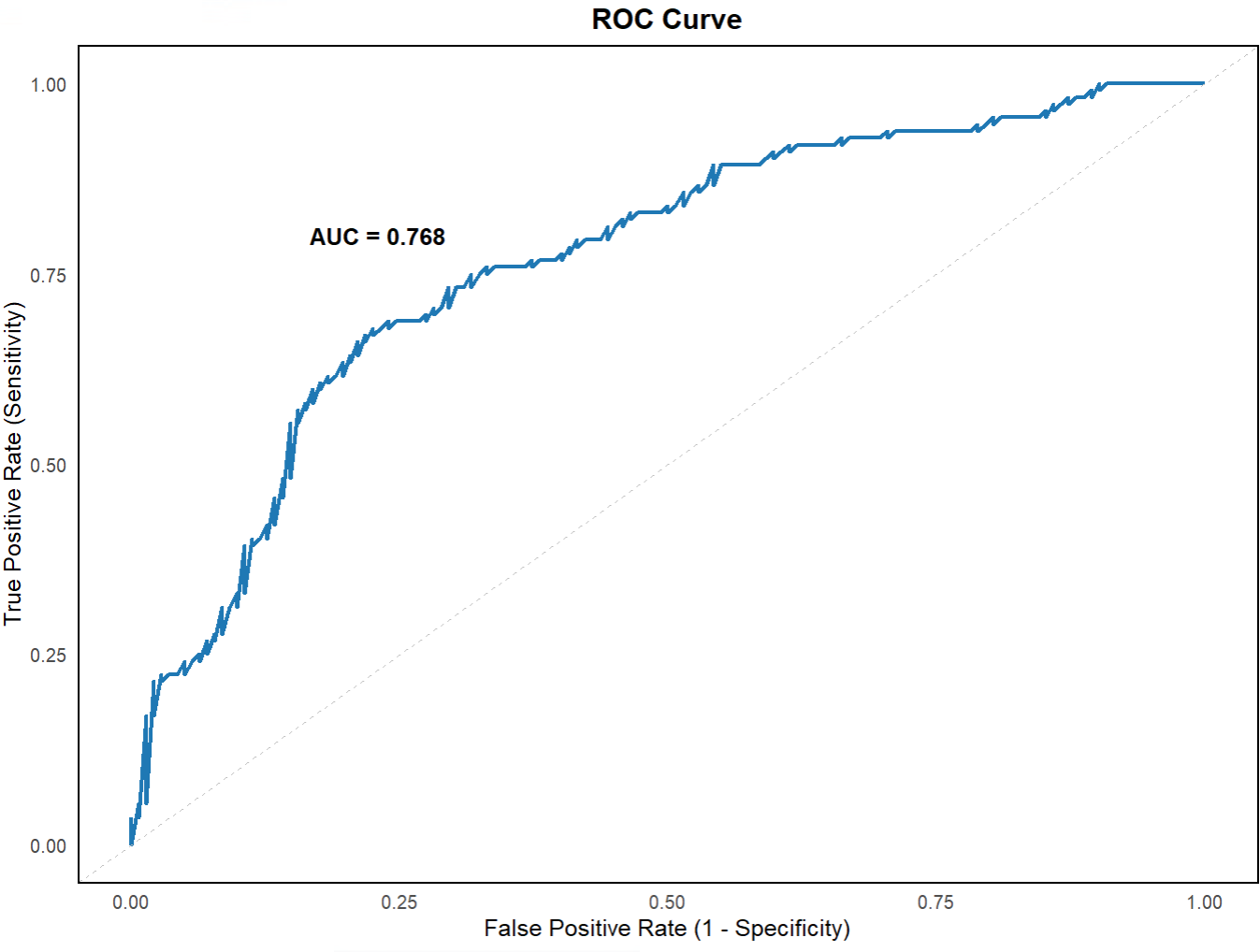


**Fig.S9**. Receiver Operating Characteristic (ROC) curve evaluating the external performance of the 2023 NYC–LI logistic model predicting *Ixodes scapularis* nymph presence, validated against 2024 Greater Boston data. The model demonstrated good discrimination, with an AUC of 0.768.


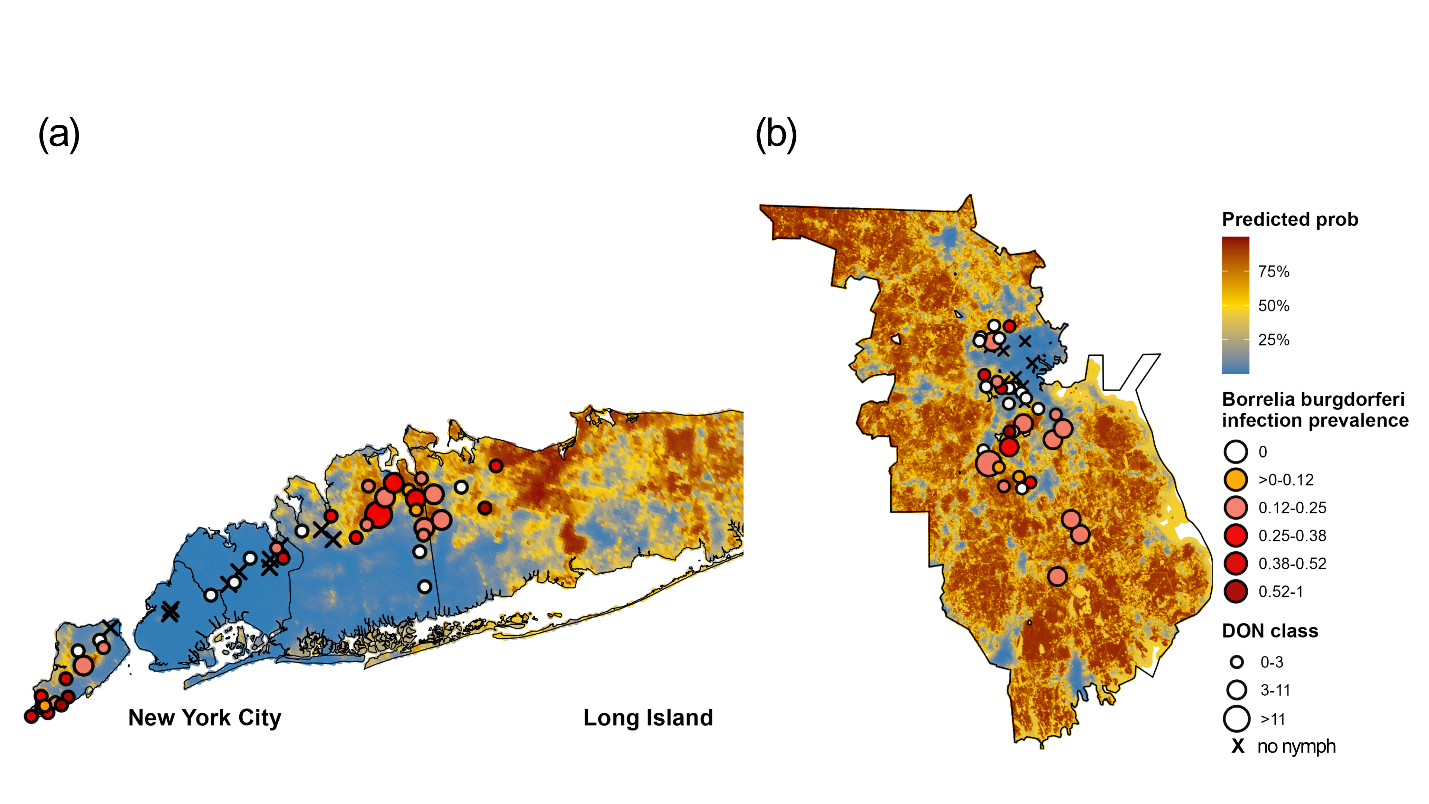


**Fig.S10.** **Predicted *Ixodes scapularis* presence probability and observed tick surveillance outcomes across New York City–Long Island and Greater Boston in 2024.** Panels show results for (a) New York City–Long Island, where 49 greenspaces were sampled, and (b) Greater Boston, where 43 greenspaces were sampled. The map illustrates the spatial correspondence between the predicted tick presence surface and independent 2024 field observations used for model validation. Background raster colors represent model-predicted probability of *I. scapularis* nymph presence, with warmer colors indicating higher predicted probability and blue colors indicating lower predicted probability. Circles indicate sampled greenspaces in 2024, with circle size representing observed density of nymphs (DON class: 0–3, 3–11, and >11 nymphs) and circle color representing observed *Borrelia burgdorferi* infection prevalence among tested nymphs. Open circles indicate sites where no infected nymphs were detected, and black “X” symbols indicate sites where no *I. scapularis* nymphs were collected.
